# Large-scale models of signal propagation in human cells derived from discovery phosphoproteomic data

**DOI:** 10.1038/ncomms9033

**Published:** 2015-09-10

**Authors:** Camille D. A. Terfve, Edmund H. Wilkes, Pedro Casado, Pedro R. Cutillas, Julio Saez-Rodriguez

**Affiliations:** 1 European Molecular Biology Laboratory-European Bioinformatics Institute (EMBL-EBI), Wellcome Trust Genome Campus, Hinxton, Cambridge CB10 1SD, UK; 2 Integrative Cell Signalling and Proteomics, Centre for Haemato-Oncology, Barts Cancer Institute, Queen Mary University of London, John Vane Science Centre, Charterhouse Square, London EC1M 6BQ, UK

## Abstract

Mass spectrometry is widely used to probe the proteome and its modifications in an untargeted manner, with unrivalled coverage. Applied to phosphoproteomics, it has tremendous potential to interrogate phospho-signalling and its therapeutic implications. However, this task is complicated by issues of undersampling of the phosphoproteome and challenges stemming from its high-content but low-sample-throughput nature. Hence, methods using such data to reconstruct signalling networks have been limited to restricted data sets and insights (for example, groups of kinases likely to be active in a sample). We propose a new method to handle high-content discovery phosphoproteomics data on perturbation by putting it in the context of kinase/phosphatase-substrate knowledge, from which we derive and train logic models. We show, on a data set obtained through perturbations of cancer cells with small-molecule inhibitors, that this method can study the targets and effects of kinase inhibitors, and reconcile insights obtained from multiple data sets, a common issue with these data.

## Abstract (web summary)

Phosphoproteomics can offer significant insight into cell signalling and how signalling is modified in response to perturbations. Here the authors develop a new tool for the analysis of high-content phosphoproteomics in the context of kinase/phosphatase-substrate knowledge, which is used to train logic models.

## Graphical abstract

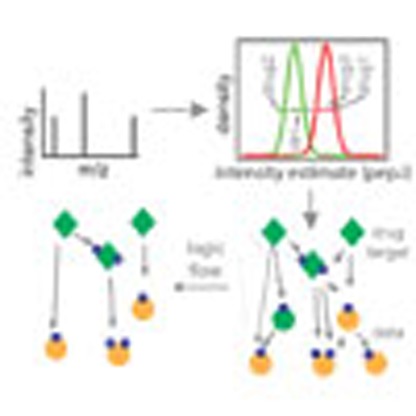

Significant technical and data-processing advances have allowed shotgun (discovery) mass spectrometry (MS), the most frequently used MS proteomics strategy, to routinely achieve a high degree of coverage of the proteome and modified (for example, phosphorylated) proteome, with ever-improving quantitative accuracy^1^,^2^,^3^. However, owing to the high redundancy and extreme complexity of proteome samples, the full spectrum of peptides present is largely undersampled in any single experiment. Hence, repeated analyses of the same or similar biological samples can show problematically low overlap of identified proteins^4^,^5^,^6^. This leads to problems of high missing-data fraction and low reproducibility, especially when using data-dependent acquisition, where simple heuristics are used to select precursors for tandem MS analysis^7^,^8^,^9^,^10^,^11^. This an be alleviated using strategies by which extracted ion chromatograms are constructed for all peptides identified in a set of samples^9^,^12^. In addition, depth of analysis comes at a high cost in terms of experimental time, which limits the ability to perform replications and analyse many conditions^5^.

Using such phosphoproteomics data (hereafter phospho-MS) data to investigate signalling by phosphorylation, we are further faced with problems linked to the specificity of kinase–substrate relationships, complexity of combinatorial and context-specific regulation, and limitations in our knowledge of both direct and indirect effects of the molecular tools used^12^,^13^,^14^,^15^. Together, these form a complex set-up with uncertainties at many levels, the like of which is increasingly successfully handled with statistical and network-modelling approaches (see for example, Ideker and Krogan^16^, and Terfve and Saez-Rodriguez^17^ for reviews). Indeed, the challenges mentioned above (uncertainty in the data, sparsity of prior knowledge), combined with a scope unmatched by other proteomics technologies, make traditional modelling approaches such as reverse-engineering and knowledge-driven model building largely unsuitable^17^. Therefore, analyses of phospho-MS to understand signalling typically result in a list of modulated abundances, of which some can be followed up on, but which fail to interrogate the connections between the elements of a signalling network, despite a clear interest from the community^2^,^8^,^15^,^18^,^19^.

In this work, we present a method (PHOsphorylation Networks for Mass Spectrometry (PHONEMeS)) to analyse changes in phospho-MS data on perturbation in the context of a network of possible kinase/phosphatase-substrate (K/P-S) interactions (Fig. 1). This method combines (i) stringent statistical modelling of perturbation data with (ii) logic model building and training based on a space of paths from perturbed nodes to affected phosphorylation sites compatible with K/P-S knowledge. Based on a phospho-MS data set acquired on the inhibition of kinases with small molecules, we show that PHONEMeS is capable of recapitulating known relationships between different perturbed kinases and their substrates. Furthermore, it organizes the data in a way that is readily interpretable as a network of regulatory relationships as opposed to a list of sites affected by the inhibition of a particular kinase. We demonstrate the power of this approach by modelling the effect of the inhibition of multiple kinases in a breast cancer cell line and verify the unexpected prediction that mTOR inhibition affects the function of the cyclin-dependent kinase CDK2. Finally, using an independent data set (obtained with the same cell line but a different set of inhibitors and instruments), we show that placing the data in context with PHONEMeS allows us to reconcile the insights obtained from two data sets that seem disparate at first sight, as is often the case with discovery MS.

## Results

### Data processing for perturbation flow modelling

**Figure 1:**
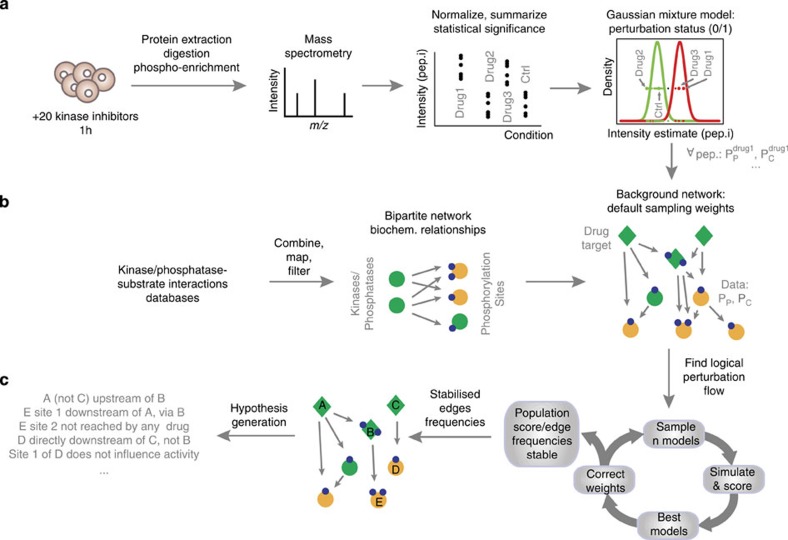
Overview of the PHONEMeS method. (**a**) Data. Cells are treated with a panel of kinase inhibitors (Supplementary Table 1), and discovery phospho-MS data are obtained. The data are normalized and a linear model used to estimate the effects (and significance) of each treatment on each peptide. A Gaussian mixture model is fitted for each peptide. Those that show a naturally Boolean behaviour with two populations (a control and a perturbed state) are selected. Each measurement (peptide, condition) is associated with the log ratio of the probability of belonging to either the control or perturbed distribution. See also Fig. 2 and Supplementary Fig. 1. (**b**) Background network. The data are mapped to a K/P-substrate network combining information from multiple databases (Supplementary Table 2) from which we extract a network of possible paths connecting drug targets and data (Supplementary Fig. 2). (**c**) Training of the networks is done by iterating through (i) sample Boolean logic models of perturbation flow; (ii) simulating the logic model to steady state; (iii) scoring based on comparison of prediction with the log ratios above; and (iv) correcting sampling weights according to frequencies of edges in best models (Supplementary Fig. 2). The resulting most likely paths (within a given tolerance) are used to generate testable hypotheses.

The data used here consist of liquid chromatography-tandem MS (LC-MS/MS) analysis of phospho-enriched proteomic extracts from MCF7 breast cancer cell line samples exposed to a panel of 20 small-molecule kinase inhibitors targeting multiple growth pathways (Supplementary Table 1) for 1 h (ref. 20). To obtain estimates of the effect of each inhibitor on each of the 12,266 unique peptides across biological duplicates and technical triplicates, as well as a rigorous measure of the reliability of these estimates, we applied a linear model with empirical Bayes shrinkage of the standard errors, followed by multiple hypothesis testing correction (see Fig. 1a and Supplementary Fig. 1a).

**Figure 2:**
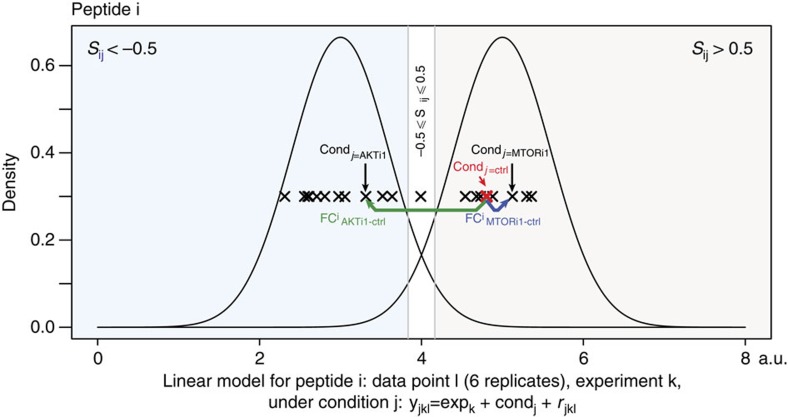
Data processing in PHONEMeS. For each peptide *i*, a linear model is fitted to estimate the effects of each condition *j*, and the significance of the fold change (FC) versus control is computed for each treatment with a moderated *t*-statistic. A Gaussian mixture model is fitted for each peptide and those that are best fitted with two components (in our pilot data, 72% of the peptides with multiple components) are kept. Each measurement (peptide *i*, condition *j*) is associated with a single number *Si,j*, the log ratio of the probability of belonging to either the control or perturbed distributions. Peptide *i* is called ‘perturbed' under condition *j* if this number is below −0.5, and it is considered to be in the control state if this number is above 0.5. Values between −0.5 and 0.5 are considered undetermined. See also Supplementary Table 3.

Boolean logic modelling is well suited for computationally efficient modelling of large-scale networks and has the potential to capture relationships between species even when the data are of semiquantitative nature^21^,^22^. However, it does make the strong assumption that the state of species observed can be satisfactorily captured with a two-level scheme. As defining a functionally relevant high/low state for each of thousands of phosphosites is unrealistic, we applied a Gaussian mixture model (GMM) on each site to determine which sites in our data sets are better captured with two distinct populations of states across the conditions observed and the location of the group of conditions where the site is at its control or perturbed state (see Figs 1a and 2). We found that 62.3% of peptides were best captured with a single component, indicating that the level of phosphorylation of sites on these peptides is not significantly affected by our kinase inhibitors. This is expected when using a discovery technology, since most of the proteome can be assumed to be unaffected by punctual perturbations such as those used here. Of those peptides that we better captured with more than one population, the majority (72.5%) showed a behaviour whereby the phosphorylation levels lie in either of two populations, one encompassing the control level and the other assumed to capture the set of conditions in which the perturbation has reached the site (Supplementary Fig. 1). We focused on these 2,376 peptides that comply with the Boolean assumption, and computed for each peptide *i* in each condition *j* a single number *Sij* (the log ratio of the probability of belonging to the control versus the perturbed distribution), which is negative when the data point is more likely to belong to the perturbed distribution, and positive otherwise (see Fig. 2). This single number can be used to derive a score that compares candidate networks based on their associated predicted states of measured nodes on perturbation (see below).

### Context-specific network building and training

The core of PHONEMeS lies in building and training a background network that represents a set of possible paths connecting inhibited kinases/phosphatases to the measured phosphosites responding to the perturbation. This aspect is conceptually similar to a previously presented methodology for low-content phosphoproteomics^23^. Manually assembling a network connecting the targets of 20 kinase inhibitors and a couple of thousand perturbed sites is obviously unfeasible. Therefore, we compiled a data set of known and predicted K/P-S interactions from multiple databases (see Supplementary Table 2), and looked for paths from perturbed K/P to data sites in this network of influence. The resulting network (hereafter referred to as ‘background network') represents the set of paths compatible with knowledge on K/P-S relationships, through which the kinase inhibitions can reach the perturbed sites (Fig. 1b and Supplementary Fig. 2a–c).

**Figure 3:**
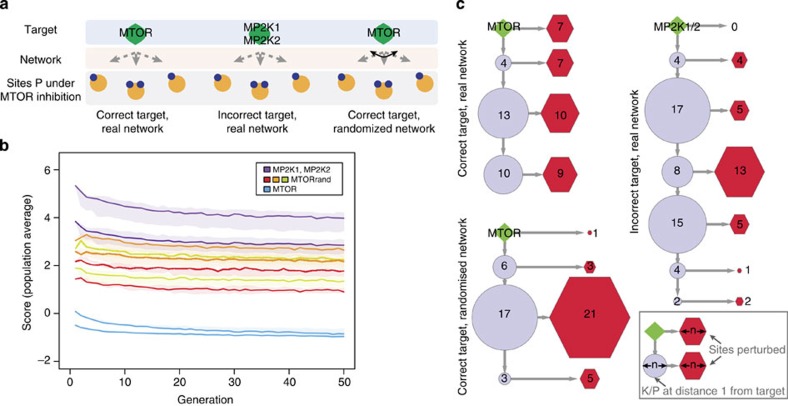
Target prediction and network information content. (**a**) We performed three sets of optimizations to model the effect of mTOR inhibition: (i) with real K/P interactions and the expected drug target (mTOR); (ii) with the real interactions but another drug's targets (MP2K1, MP2K2); and (iii) with three separate randomized sets of K/P interactions and the expected target. (**b**) Population average scores for each of the two mTOR inhibitors. (**c**) Simplified representation of the resulting networks (20% tolerance; full networks shown in Supplementary Fig. 6). The size of the nodes reflects the number of K/P or sites perturbed, at a distance *d* from the drug target. Real network/correct target: 33 out of 34 perturbed nodes connected to mTOR, average shortest path length of 4.3; 92 nodes in total. One of the three randomized networks/real target: 30 out of 34 perturbed nodes connected to mTOR, average shortest path length of 4.9; 83 nodes in total. Real network/incorrect target: 30 out of 34 perturbed nodes connected to MP2K1/MP2K2, average shortest path length of 8.1; 141 nodes in total. Most sites are connected to the designated targets in all cases but the paths are shorter with the correct target than with the incorrect ones. The random networks show a major difference in terms of number of sites explained directly by the drug target or its first neighbours (14 sites for the real network versus 4 sites for this random network).

Training is done by iteratively sampling and scoring candidate networks based on evidence from the data (see Fig. 1c and Supplementary Fig. 2d–g). Candidate logic models are built from the space of solutions compatible with knowledge, by sampling incoming hyperedges (that is, sources and their logic combination) for each node in the network (Fig. 3d). Each candidate network is then simulated until a steady state is reached under all conditions considered, and the predicted and observed states of nodes are compared (Supplementary Fig. 2e) by computing a score


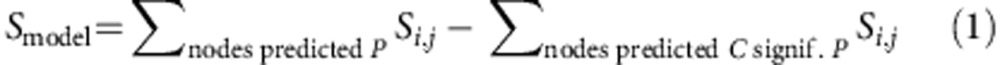


that rewards correctly predicted perturbations and penalizes wrongly predicted and missed perturbations. Based on these model scores, we select a family of best-performing models, and update the sampling procedure accordingly (Supplementary Fig. 2f–g). Training finishes when the average score and sampling frequencies of edges for the population of models sampled in each generation stabilize. Multiple independent training results (that is, frequencies of edges in the final population of models) are typically combined into a single solution to account for the stochastic nature of the optimization. The results are visualized as a single network where each node is represented with its highest frequency inputs with a user-defined level of tolerance. The tolerance (edges within *x*% of the highest frequency input) is used to assess the extent to which different areas of the network are constrained by the data.

### Target prediction power and network information content

Having established the validity of the method using a proof-of-principle analysis (see Supplementary Note 1 and Supplementary Figs 3 and 4), we wanted to determine (i) whether PHONEMeS had the ability to discriminate incorrect targets and (ii) whether combining data with knowledge in the form of a network structure brings valuable information to the training process. Indeed, when looking at paths in the K/P-S network to the sites found in our data to be perturbed under inhibition of the kinase mTOR (mammalian target of rapamycin), we found that there are two groups of K/P: those that can reach all perturbed sites for a condition, and those that cannot reach any (Supplementary Fig. 5). Therefore, any K/P in the first group would be a viable drug target candidate in the sense that they have potential paths to all sites perturbed under that drug treatment.

To answer these, we compared three analyses aiming to capture the effects of mTOR inhibitors (Fig. 3a). In the first setting, we built and trained models attempting to connect the correct drug target (mTOR) to the sites perturbed under mTOR inhibition. In the second setting, we instead used the targets of another drug (MP2K1 and MP2K2, targets of the MEK inhibitors), and in the third setting we used the right target but randomized K/P-S networks (obtaining by randomly shuffling the K/P across K/P-S interactions). As we can see from the scores plots (Fig. 3b), networks based on the wrong information (wrong targets) provide clearly worse fits (pink–purple curves) than those based on plausible targets (blue–turquoise curves). The random network scores (green–red curves) cover a range between these two extremes. These results suggest that an optimization with a biologically realistic network but with the incorrect targets performs worse than a random network, which itself performs worse than a realistic network with the right target. These results would presumably be influenced by the specificity of the drug and the similarity of the neighbourhoods of candidate targets. However, at least in this case study, our analysis indicates that prior knowledge, at both the drug target and network level, is informative as it leads to better fits to data. Looking at the resulting networks (Fig. 3c and Supplementary Fig. 7), we can see that even though all settings can connect chosen targets to most sites, the incorrect targets reach the perturbed sites through longer paths, with an average shortest path length of 4.3 with the real target and 8.1 with the wrong target. Comparing the real and randomized networks, the biggest difference lies in the number of sites that are found directly under the drug target or one of its first neighbours (mTOR, AKT1, KS6B2/KS6B2 and SGK1 cover 14 sites in the real network, whereas mTOR and its first neighbours only explain 4 sites in the random network). This indicates that, when working with kinase inhibitors, one should question the assay and assumed drug targets when none of their known direct substrates are affected. In summary, this analysis indicates that considering the scores and resulting networks (proximity of sites to drug target, simplicity of the network, etc) should allow us to prioritize drug targets as being more or less likely. Accordingly, we have used PHONEMeS to analyse the effect of each of the 20 drug treatments applied in this data set (see Supplementary Note 2 and Supplementary Table 7). This allowed us to get a novel perspective on the specificity and efficacy of kinase inhibitors.

### Modelling the effect of mTOR inhibition

**Figure 4:**
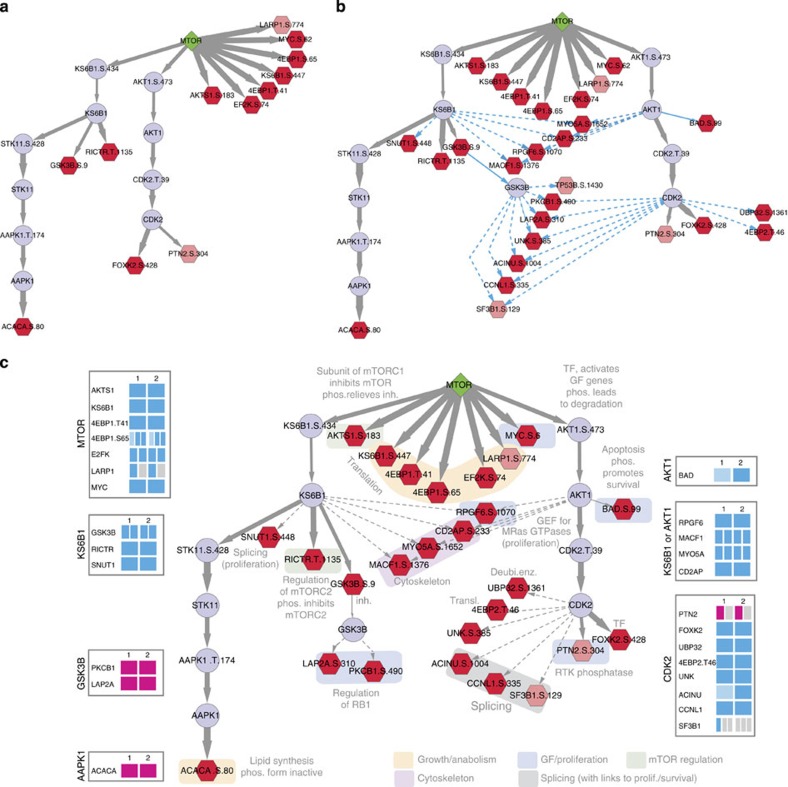
Modelling the effect of mTOR inhibition. (**a**) Fifteen sites perturbed under one of the two mTOR inhibitors in our data were connected to mTOR in three independent optimizations, using exclusively experimentally demonstrated interactions. Results of these were combined into average frequencies in final populations of models, which were used to extract a best-consensus model with 20% tolerance around the highest frequency edges. This model recapitulates effects on known substrates of mTOR, and highlights a good agreement between the effects of the two inhibitors. (**b**) Sites that were found to be perturbed but did not have an experimentally demonstrated kinase were connected to the optimized network based on predictions from networKIN. (**c**) Predictions were prioritized based on the sign of the perturbations observed, and functional annotations of proteins were extracted from Uniprot. This representation allows to analyse such data in a way that directly traces the effect of a kinase inhibition on the phosphoproteome, and prioritizes predicted kinases.

We used PHONEMeS to model the propagation, through the phospho-signalling network, of mTOR inhibition with two distinct mTOR inhibitors (Fig. 4 and Supplementary Table 1). The mTOR kinase interacts with multiple proteins to form two complexes: mTORC1 (which responds to nutrients and regulates processes such as protein synthesis and autophagy) and mTORC2 (which responds to growth factors). The regulation and downstream effects of mTOR in either of these are complex and only partially characterized^24^, but potent specific inhibitors of mTOR as the catalytic subunit of both complexes are available. Indeed, each of the inhibitors used here result in 37 significantly perturbed phosphosites, of which 32 are common, indicating both efficacy and common specificity. Connecting these sites to mTOR in the network of possible K/P solutions results in a network of 311 nodes and 4,859 edges (Supplementary Fig. 6). After applying PHONEMeS excluding predicted interactions, we obtained a trained network that is easily visually interpretable, comprising 30 nodes and 32 edges with 20% tolerance (Fig. 4a). This network shows that mTOR inhibition results in an expected decrease of phosphorylation of many canonical mTOR substrates (for example, MYC, 4EBP1, AKTS1, KS6B1). It also shows a propagation of the signal further downstream through an effect on KS6B1 and AKT1. Therefore, PHONEMeS successfully placed the perturbation data in context, resulting in a noticeable increase in interpretability. Because many of the sites affected by mTOR inhibition in our data do not have experimentally demonstrated kinases, we wanted to see if we could use our trained network to confirm which of their predicted kinases is most likely to be responsible for their phosphorylation in this context (Fig. 4b,c). Based on their predicted kinases, we were able to connect an additional 14 perturbed sites to the perturbation of mTOR, and to provide strong evidence in favour of 1 (or 2) possible kinases among the 5 to 16 kinases that were predicted for these sites (see Supplementary Table 5). Finally, functional annotation of the resulting network (Fig. 4c) allowed us to explicitly show effects of mTOR inhibition on downstream processes such as translation, regulation of the cell-cycle/survival, and interactions within the two mTOR pathways.

### mTOR effects CDK2-mediated G1/S-phase transition

**Figure 5:**
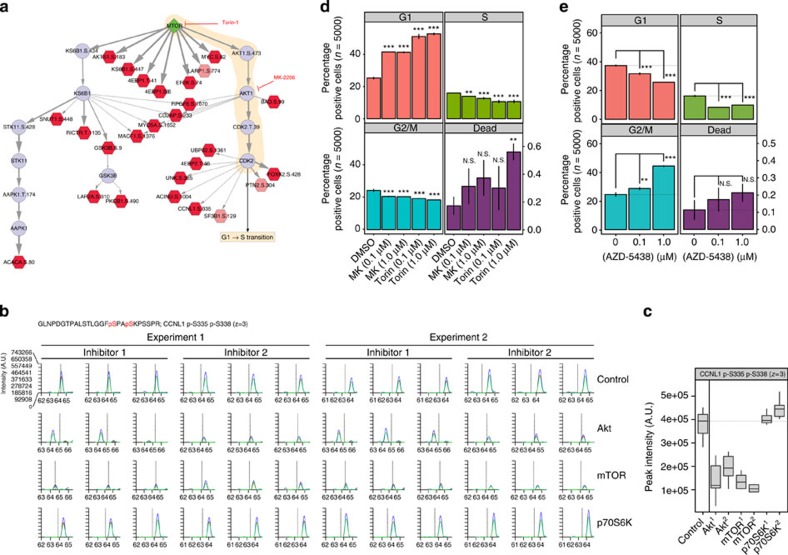
Inhibition of mTORC1/2 or Akt prevents CDK2-mediated G1- to S-phase transition. (**a**) The prediction that Cyclin-L1 phosphorylation is affected by mTOR inhibition via Akt and CDK2 was tested by inhibiting Akt and mTOR in a validation experiment. (**b**) Extracted ion chromatograms for the ion representing two phosphorylation sites on Cyclin-L1 (CCNL1) under treatment with mTOR, Akt and P70S6K inhibitor treatments confirm the topology predicted in **a** (blue lines, 1st isotope; red lines, 2nd isotope; green lines, 3rd isotope). (**c**) Box plots summarizing the raw intensity data shown in **b**. Dotted line represents the median intensity under treatment with DMSO vehicle control. (**d**). Cell cycle assays for MCF7 cells treated with DMSO, MK-2206 (Akt inhibitor) or Torin-1 (mTORC1/2 inhibitor) show an increase in cells in G1 phase and a decrease in cells present in S and G2/M phase on treatment, consistent with an alteration of the activity of CDK2. (**e**). Cell cycle assays for MCF7 cells treated with DMSO or AZD-5438 (CDK1/2/9 inhibitor) show an increase in cells in G2/M phase and a decrease in cells in G1 and S phase on treatment, consistent with an effect on CDK1. P-values derived from one-way ANOVA and adjusted for multiple testing (***P<0.001; **P<0.01; NS, not significant).

The above analysis predicted an interesting path between mTOR and CDK2 (via Akt), leading to perturbation of the phosphorylation level of predicted substrates of CDK2 such as cyclin-L1 (CCNL1) (see Fig. 4c). Therefore, we wanted to verify both the plausibility of this predicted path to CCNL1, and the hypothesis that mTORC1/2 inhibition leads to a perturbation of the activity of CDK2. First, we constructed extracted ion chromatograms for the ion shown in Fig. 5b, which covers the Ser^335^/Ser^338^ phosphorylation sites (*m*/*z* 891.0787; tR≈64 min) across the control and drug-treated samples (see also Supplementary Fig. 9). As we can see in Fig. 5b,c, this phosphopeptide was decreased 2–5 fold in abundance following Akt and mTORC1/2 inhibition, but not following P70S6K inhibition, confirming that the path mTOR→AKT→CDK2 is likely to be responsible for the fact that perturbation of mTOR reaches CCNL1. This site was also inhibited by PI3K inhibitors, but not by the other inhibitors tested (see Supplementary Fig. 9). Given that the sequence flanking the CCNL1-Ser^335^ phosphorylation site shows a consensus CDK2 phosphorylation motif (xSPxxK^25^), it is likely that this site is the CDK2 substrate of which phosphorylation level is affected by mTOR inhibition in a mechanism that is Akt and CDK2-dependent.

Next, we wanted to verify that the cell biological effect of CDK2 is indeed altered when mTOR and Akt activities are modulated, as the PHONEMeS prediction suggests. CDK2 is known to control the transition of cells from G1 to S phase^25^, such that inhibition of CDK2 activity leads to a stalling of the cells in a G1–G0-like state due to an inability to make the transition into S phase. Therefore, if our predictions are correct, inhibition of mTOR/Akt should result in a decrease of CDK2's activity (as evidenced by the decrease in phosphorylation of its substrates as shown in Fig. 5a), and therefore an increase in the proportion of cells unable to transition into S phase and beyond. To test this, we treated MCF7 cells with specific Akt and mTORC1/2 inhibitors for 24 h (see Fig. 5a) and subsequently measured the proportion of cells in each cell-cycle phase by flow cytometry (see Fig. 5d). As we can see in Fig. 5d, on pharmacological inhibition of either Akt or mTORC1/2, the proportion of cells present in G1 phase increased approximately 1.7- and 2-fold, respectively, relative to DMSO control, and the proportion of cells present in S and G2/M phase was concomitantly reduced. By contrast, on treatment with a CDK1/2/9 inhibitor (Fig. 5e), the proportion of cells in G2/M phase was increased, consistent with an effect on CDK1 and confirming that the effect observed on Akt and mTOR inhibition is likely to be CDK2-dependent and CDK1-independent. As a whole, these data suggest that inhibition of either mTORC1/2 or Akt reduced the biochemical (Fig. 5b,c) and functional (Fig. 5d) activity of CDK2, thus giving support to the predictions from the network model (Fig. 5a).

### Reconciling insights from independent data sets

**Figure 6:**
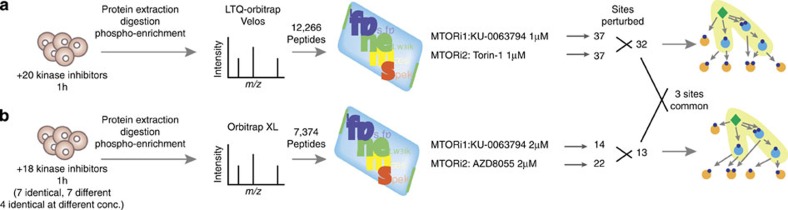
Putting phospho-MS data in context: set-up. This analysis aims to investigate the potential of PHONEMeS to reconcile insights obtained from two similar data sets despite the actual sites sampled being widely different. We separately analysed two similar data sets (**a**, **b**) obtained in two different laboratories, with a partially overlapping set of small-molecule kinase inhibitors. We focused on the pairs of drugs with mTOR as nominal target because out of the six nominal targets in common between the two data sets, the mTOR inhibitors appeared to be the most specific drugs with a clearly detectable effect. The data sets have widely different coverage of the phosphoproteome, and only show three sites in common among the sites that are perturbed on mTOR inhibition.

**Figure 7:**
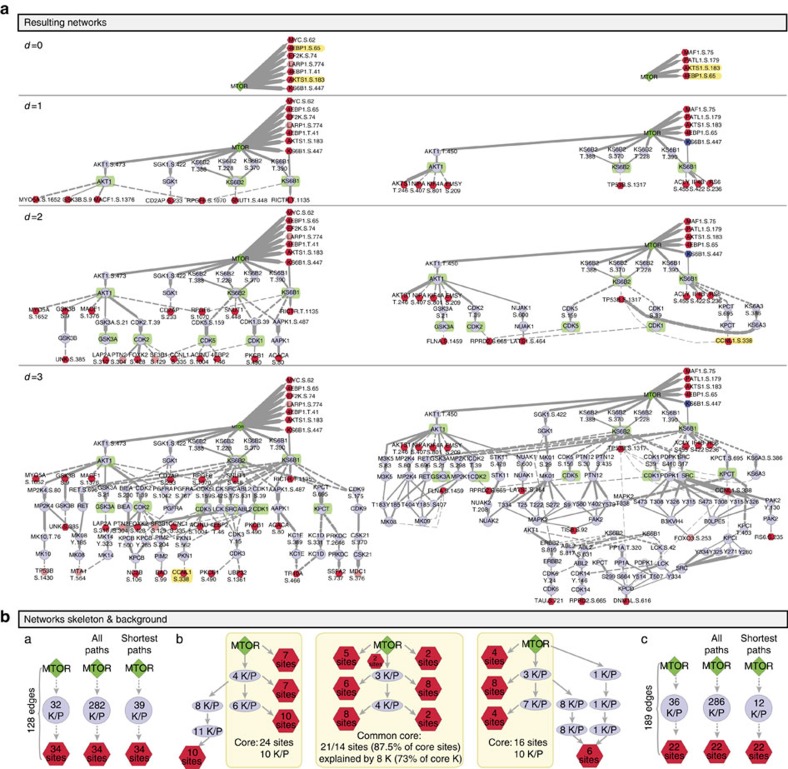
Putting phospho-MS data in context: results. (**a**) Networks resulting from independent analysis of two separate data sets on mTOR inhibition, laid out by distance from the drug target (mTOR) (kinases highlighted in green and perturbed sites in yellow are identical between the two networks). (**b**) Structure of the resulting and background networks. (a,c) Structure of the background networks for the main (a) and the validation data set (c). As expected (see Supplementary Fig. 4), the number of K/P that can reach perturbed sites from mTOR are roughly equivalent in the two data sets (a versus c, left), but the minimum number of K/P necessary to connect these (b versus d, right) is much smaller in the validation data set (c), which contains less sites to connect. The results from the validation data set are however less constrained (higher number of edges within tolerance), presumably as a result of shallower sampling of the proteome. (b) Structure of the 20% tolerance resulting networks for the two data sets. The core networks (paths with evidence at every step) explain, respectively, 4 and 16 perturbed sites with 11 kinases. Eight of these kinases are common between the two analyses, and explain 87.5% of the sites in the core network of each analysis, despite the overlap in sites identified consisting of only two sites. Comparing the resulting and background networks, we can see that the training to data allowed to strongly constrain the background network, but solutions do not simply represent the shortest possible paths.

A fundamental problem with discovery MS lies in the poor agreement between multiple, biologically similar, data sets^7^,^11^. We reasoned that if this was mostly a result of the undersampling problem^4^,^10^, then placing the data in its molecular context should be able to reconcile the insights obtained, even if the individual sites that are sampled in each data set are different. To test this hypothesis, we applied PHONEMeS on an unpublished, novel but related data set that was obtained in a different physical location, on a different LC-MS/MS instrumentation and with a partially overlapping set of drugs (Fig. 6 and Supplementary Table 4). The two data sets differ substantially, both quantitatively (depth, that is, number of peptides measured) and qualitatively (sites perturbed identified, drugs used), as summarized in Fig. 7. However, when we put the two networks obtained in parallel (Fig. 7), we can see that the insights are remarkably similar. Indeed, both resulting networks consist of a ‘core network' (connecting 24 out of 34 and 16 out of 22 sites, respectively), with paths of length up to two kinases from mTOR supported by perturbed sites at every step, and a ‘peripheral network' containing exclusively paths of three kinases, comprising kinases not supported by data and many predicted edges. Even though the sites sampled in each core network differ (only 2 sites are common, out of 24 and 16 sites, respectively), we can see in Fig. 6b that eight kinases that are common between the two networks explain 87.5% of sites in the core network. Discrepancies in the ‘peripheral networks' presumably result from a decreased quality of knowledge in the relationship between the target and distant sites as we get further away from mTOR, as well as a decreased intensity and reliability of the biological signal. Importantly, the kinases selected in the common core network are not simply those that form the shortest path between sites perturbed and mTOR (Fig. 6b). Indeed, respectively 24 and 9 of these 39/12 shortest paths K/P are actually chosen for each data set, combined with an additional 8 and 27 K/P. In conclusion, out of a possible 282/286 kinases lying on paths linking mTOR to its perturbed substrates in each data set, PHONEMeS shows that a remarkably similar set of kinases best explains these two *a priori* disparate data sets independently (Fig. 6b).

To confirm the general applicability of PHONEMeS, we used the approach to analyse a published mTOR inhibition data set^26^, generated using a different cell line and a very different MS approach, that is, iTRAQ labelled data with a very limited number of replicates and conditions, and a lower proteome coverage. We found that our approach both confirmed our own insights about mTOR inhibition and allowed to generate new insights that could not be obtained in the original study (see Supplementary Note 3 and Supplementary Fig. 10).

## Discussion

In this paper, we introduce a method, termed PHONEMeS, which places discovery phosphoproteomic MS data on perturbation in the context of a network of possible K/P-S interactions. This method reconstructs paths from kinases inhibited on drug treatment to sites perturbed by these inhibitions, using a Boolean logic modelling. The assessment of whether a site is perturbed, as well as the scoring of models based on comparison between data and model prediction, is based on a stringent multi-step probabilistic scoring scheme. We found that this method is capable of discriminating incorrect targets from kinases that are truly affected by a drug treatment, which is extremely valuable given that the *in vivo* specificity of kinase inhibitors is often poorly defined^15^. In addition, we observed that the data-driven training results in a considerable decrease in the complexity of the networks that have to be interpreted, compared to the full network of paths compatible with prior knowledge. Finally, we demonstrated the use of this method as a means to place discovery MS data on perturbation in a clearly and easily interpretable context, leading to an easily testable hypothesis. We went on to validate some of these predictions, such as a predicted phosphorylation of CCNL1 by CDK2, leading to perturbation of the phosphorylation of CCNL1 on inhibition of mTOR and Akt but none of the other kinases in the network. We additionally validated the prediction that CDK2's activity is perturbed by mTORC1/2 and Akt inhibition, resulting in an alteration of the proportion of cells in the G1, S and G2/M phases of the cell cycle. We also showed the use of PHONEMeS to compare insights from multiple similar data sets despite these having little overlap at first sight, a common problem with discovery MS.

Other studies have looked for paths in large networks, mostly using protein–protein interaction (PPI) networks and different types of experimental data^27^,^28^,^29^,^30^,^32^. Because of the nature of the networks that frequently support these procedures (that is, PPI networks or compendium functional networks such as STRING^33^), the resulting networks connect experimentally or knowledge-derived ‘hit nodes' rather than physically interpretable and testable paths, and do not include site-specific information. In addition, there rarely is a clear and consistent physical interpretation of paths, because the search procedure is often designed to find minimal paths of influence or minimally connecting subnetworks, and is agnostic to the biological interpretation of edges, which is often widely heterogeneous. Furthermore, many of these approaches ignore quantitative information associated with ‘hit' nodes that they aim to connect, and impose some constraint on inclusion of nodes without data, thereby penalizing missing data/data with high false-negative rates and incomplete coverage. This is a reasonable choice when using genomic and transcriptomic technologies where coverage is less of an issue, but less so with respect to proteomics experiments. Finally, many of these methods cannot simultaneously capture multiple conditions (and disjoint sets of associated hits) in a single network. Therefore, multiple conditions require either searching for multiple separate networks or lumping all nodes to be connected as if they originated from the same perturbation. Some studies^34^,^35^,^36^ have applied well-known reverse-engineering approaches (for example, correlation-based), but using extensively manually curated data of much smaller scope. A few studies^12^,^37^ have used very similar data to ours but their insights were focused on inferring fragments of a phosphorylation-based network, in the form of kinase–substrate relationships and individual kinases likely to be affected by a perturbation, rather than retrieving global paths of perturbation as we do here. Our study therefore demonstrates a novel approach to derive biological information from phosphoproteomics data that can complement existing tools and that, crucially, can provide insights not attainable with current approaches.

A number of assumptions underlie our approach: first, since it constrains the space of solutions to what is compatible with known and predicted K/P-substrate specificity, anything outside this scope cannot be found (although candidate interactions can easily be added prior to training). This allows constraining solutions to an *a priori* realistic set, which seems like a reasonable compromise when working with very high-content (but comparatively low-sample-throughput) data. Second, given the nature of regulation by phosphorylation, the background networks tend to be very complex, sometimes resulting in poorly constrained (or non-identifiable) areas. This limits the insights that can be generated from the analysis for certain sections of the network, a feature that we make visible in the solutions. Ongoing developments in high-content MS technologies should make rich experimental designs more attainable, resulting in more constrained models. Third, our approach describes rapid change on perturbation (mathematically assumed to correspond to a pseudo steady state) based on data at a characteristic time point on perturbation. This is mostly a decision associated with the limited availability of perturbation time-series data and the complexity of constraining both structure and dynamics on such large scales. Modelling change on perturbation implies that the edges do not capture activity *per se*, but a change in a K/P's activity on perturbation, at the time of measurement. Finally, our work uses phosphorylation as a proxy of alteration of activity. Hence, the presence of a path does not necessarily mean that phosphorylations caused the activation/inhibition cascade, but that they could occur concurrently with those events. Conversely, an activation/inhibition path could simply be absent if no phosphorylation path could be found that co-occurs with the cascade. Other available data (for example, about other post-translational modifications) could easily be included in our framework to address this.

This approach represents a step forward in the functional, context-specific interpretation of discovery MS phosphoproteomic data on perturbation, such as but not limited to kinase inhibition: any perturbation that can be connected to the K/P-S network (genetic, extracellular ligand and so on) can in principle be used. We are planning to investigate several additional features for this method, such as different ways to (i) include (and study) the logic combinatorial complexity in the network (which we have largely ignored here because, without combinations of perturbations, complex logic gates rarely reach high frequencies); (ii) include multiple states of regulation, such as for example, control/up/down; (iii) extend the background knowledge to interactions based on lipid phosphorylation and phospho-binding domains.

## Methods

### Data generation

The main data set used in this paper is described in Wilkes *et al.*^20^. Briefly, cells are treated for an hour with one of 20 small-molecule kinase inhibitors (see table ST1) or the DMSO vehicle. Cells are collected, lysed and enriched for phosphopeptides using TiO2, and samples are run through an LC-MS/MS (LTQ-Orbitrap-Velos) in (technical) triplicates. Each treatment is performed in biological duplicates (successive early passages), leading to a total of six replicate measurements for each peptide and drug treatment. The peptide identification is done using Mascot and the quantification is done using the Pescal software^38^, providing peak heights from extracted ion chromatograms for each phosphopeptide ion. For the second data set, the analysis pipeline is identical except for the inhibitors used (see table ST4) and the mass spectrometer (LTQ-Orbitrap-XL). The construction and visualization of extracted ion chromatograms (XICs) was automated with a computer programme written in Python that interfaces with MSFileReader (ThermoFisher Scientific) as reported previously^9^. The time and mass windows for the generation of XICs were 1.5 min and 7 p.p.m., respectively.

### Cell-cycle analysis

MCF7 cells were plated in six-well plates at a density of approximately 0.5 × 10^6^ cells per well in Dulbecco's modified Eagle's medium (supplemented with 10% foetal bovine serum; 1% penicillin/streptomycin). Following a 24-h incubation period, these cells were treated with 0.1% DMSO, 0.1 or 1.0 μM AZD-5438, 0.1 or 1.0 μM MK-2206, or 0.1 or 1.0 μM Torin-1 for 24 h. Following this incubation, the cells were washed with PBS, trypsinized and collected by centrifugation (450*g*, 5 min). Cells were fixed with the drop-wise addition of 10 ml ice-cold 70% EtOH—with constant agitation—and incubated at 4 °C for 2 h. Fixed cells were then washed with PBS, collected by centrifugation (as above) and stained in the dark with 0.7 ml Guava Cell Cycle reagent (Merck Millipore, USA). Stained cells were then measured through the use of a Guava easyCyteTM Flow Cytometer (Merck Millipore, USA). Each sample was run in triplicate on the instrument and 5,000 cells were analysed in each replicate injection.

### Data processing

The raw peak heights were log transformed and quantile normalized, and a linear model was applied to estimate the effect of each treatment and experimental artefact, as implemented in the Bioconductor package limma. Multiple designs were tested and a design with a factor for each drug, a single control and a factor for the biological replicates (experiments were done in two sets, each containing a control and a set of drugs, and two biological replicates) was selected based on Akaike's Information Criterion. The linear model was fitted taking into account the inter-technical replicate correlation. We then estimated the log fold change between each kinase inhibition and the control condition for each peptide, and computed the significance of this fold change using a moderated *t*-statistic by empirical Bayes shrinkage of the standard errors. The resulting *P*-values were corrected for multiple hypothesis testing using a Benjamini–Hochberg procedure applied on each condition, as implemented in the R function p.adjust. We fitted a GMM on the estimates from the linear model, separately for each peptide, across 21 conditions (20 drugs, 1 control), using the R package mclust. The package implements an expectation-maximization algorithm to fit a GMM with one to nine components and return the optimal model according to a Bayesian information criterion. Of the 11,654 peptides for which a model could be fitted, 7,263 were best fitted with one component, 3,183 with two, and 670 with three. We filtered the data to exclude cases where the areas under the density curves of the two distributions overlap by more than 10% and excluded those peptides for which the control intensity could not be estimated due to insufficient data. A total of 2,376 peptides fulfilled all of these conditions. Based on the Gaussian parameter estimates obtained for the 2,376 peptides mentioned above, we computed a single number for each site *i*/condition *j*: *S*_*i,j*_*=*log_*10*_(*P*_*i,j*_(*C*_*i*_)/*P*_*i,j*_(*P*_*i*_)) where *P*_*i,j*_(*C*_*i*_) is the probability that peptide *i* in condition *j* belongs to the control distribution for peptide *i* and *P*_*i,j*_(*P*_*i*_) is the probability that peptide *i* in condition *j* belongs to the perturbed distribution for peptide *i*. This number is negative when the measurement is more likely to belong to the perturbed distribution, and positive otherwise.

### Background network

We assembled the K/P-S data from multiple databases (Phospho.ELM, PhosphoSitePlus, HPRD, NetworKin and DEPOD, all downloaded in January 2013), mapped it to Uniprot identifiers (UPIDs) and filtered it to contain only reliable, human protein–protein information. The NetworKIN data were filtered to exclude interactions with either a motif or context score below 0.5, and predicted interactions for CSK21 and CSK22 (2288 each) were excluded because of low specificity. The final bipartite K/P-S network contains 128,251 interactions between 604 kinases/phosphatases and 17,319 sites on 4,567 proteins (see ST2). As discussed, this exclusively contains PPIs. Additional types of knowledge can be added to the background network and be subjected to training in an identical manner. This is the case, for example, for the link between PI3K (PK3CA, D) and PDPK1.S393, which is added to the knowledge to reflect the activation of PDPK1 as a consequence of lipid phosphorylation by PI3K. A background network is built from this graph reflecting two main goals: (i) finding relationships between perturbation targets, and (ii) finding kinases/phosphatases that are affected by the perturbations and are responsible for the altered phosphorylation levels in the data. Because the altered sites change from data to data, background networks are specific to each data set. First, we extract a directed network at the protein level that connects drug targets, including paths of up to seven nodes. Second, we collect all K/P that potentially target our data sites. Finally, we collect all K/P in the second network to all K/P in the second network, with paths of up to five nodes at the protein level. We then translate PPIs back into their protein-site equivalents, and add ‘integrator edges' to bridge the site-protein steps in protein–protein paths (see SF2). All nodes in the background network are recorded with their UPIDs, and UPIDs (often without the ‘_HUMAN' suffix) are used to refer to nodes in starting and resulting networks.

### Iterative training to data

*Sampling*. Logic models are sampled from the background network at each generation. Sampling is done for each node independently by dividing the (0,1) interval into as many bins as allowed, with input combinations of edges and drawing from the uniform distribution. For integrators, the allowed input combinations are as follows: each edge independently, all edges together as a single AND or no edge. For example, an integrator with two incoming edges has four input combinations: (edge1, edge2, edge1 +edge2, no edge), each getting one of four bins, that is, a probability of 0.25. For a sink node, we sample a single K/P (for example, for a sink with two incoming edges: (edge1, edge2) each gets one of two bins, that is, a probability of 0.5). For intermediate nodes, we allow all possible logic combinations of input edges by drawing for the edges and then separately for the gate that will combine them. For example, considering an intermediate with two incoming edges (edge1, edge2) are sampled independently with 0.5 probability, and the AND/OR is sampled with 0.5 probability. As a result, both edges will be sampled simultaneously in an expected 25% of models (half of which will are expected to be sampled with an ‘AND' gate), no edge will be sampled in 25% of models (expected), and either edge only will be sampled in half of the remaining 50% of cases each. These add up to the following possibilities: (edge1, edge2, no edge, edge1‘AND'edge2, edge1‘OR'edge2), with respective probabilities (0.25, 0.25, 0.25, 0.125, 0.125) (more details in SF2).

*Scoring*. Models in the sampled population are simulated under each condition (drug or identical set of drug targets) by deterministically propagating perturbations from drug target nodes in the Boolean logic model, until reaching steady state. The score of a model is obtained by summing for each simulated condition over the *j* data conditions that map to the set of drug targets used in the simulation (that is, evidence from multiple drugs with the same targets are added up):


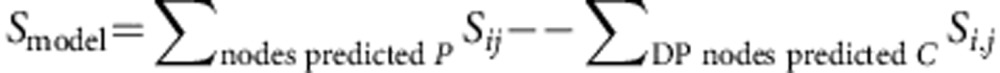


(that is, true positive predictions (negative *S*_*i,j*_)+false-positive predictions (positive *S*_*i,j*_)−false-negative predictions (negative *S*_*i,j*_)). The best models are associated with the lowest (most negative) scores. True negative predictions are not taken into account because with discovery (untargeted) MS data we expect negative measurements (nodes not perturbed) to outweigh positive ones under any particular condition. Note that the *S*_*i,j*_ are obtained by peptide, and the simulation gives perturbed/control information at the site level, so when multiple peptides match to the same site, their *S*_*i,j*_ are added up as independent pieces of evidence. A family of best models (with a user-defined level of tolerance around the best model of the iteration) is selected and used for weights correction.

*Weights correction*. This is done by virtually copying edges a number of times that is proportional to their frequency in the best models, with a fixed ceiling (‘cap' parameter). For the integrator example above, if the ceiling is cap=2 and edge1 is present in 50% of best models, and the AND in the other 50%, then the new possibilities are (edge1 × 2, edge2, edge1 +edge2) × 2, no edge), with respective probabilities (2/6, 1/6, 2/6, 1/6). For the sink example, if edge1 is present in 100% of best models, then the new possible inputs are (edge1 × 3, edge2), with respective probabilities (0.75, 0.25). For the intermediate example, if edge1 is present in 100% of best models, and edge2 is present in 50% of best models, with the AND gate in 50% of best models, then edge1 and edge2 will be sampled with respective probabilities 3/5 and 2/5, and the AND will be sampled with probability (0.5+0.5)/2 (average of the previous sampling weight and the frequency of AND in the best models of the generation).

*Results analysis*. The iterative training is finished when the average score of the population of sampled models and the frequency of edges in this population stabilize. These frequencies are the final result of a single optimization, and they are normally combined and averaged across multiple independent optimizations to account for the stochastic nature of the optimization. The results of combined optimizations are expressed as a ‘majority voting model', which is the model where each node has as input the edge with maximum (averaged) frequency in the trained populations. Starting from this single ‘consensus' model, we look at a spectrum of models with increasing levels of noise, where each node receives all edges with a frequency within a certain percentage of the best input frequency. All of the computation is done in R, and the resulting networks and attributes are output as flat text files that can be directly imported and visualized in Cytoscape^39^.

All the code is freely available as an R package under licence GPLv3, as well as tutorials and Cytoscape files on http://www.cellnopt.org/PHONEMeS

*Proof-of-principle and influence of parameters*. For these analyses (Fig. 3), three independent rounds of optimization were performed for each setting, with parameters set to the following: direct interactions=G1, cap=5, *n*=5,000, tolerance=30%, sizeP=1, unless stated otherwise. Optimizations were run for 50 generations, at which point all had stabilized (see Supplementary Fig. 3).

*Target discrimination and random networks*. For these analyses (Fig. 3), the data relating to the two mTOR inhibitors in Supplementary Table 1 (sites perturbed under either drug) were used as a basis to build and train background networks using (i) the real K/P-S bipartite network and MTOR_HUMAN as a target; (ii) the real network and MP2K1/MP2K2_HUMAN as targets; and (iii) three different randomized versions of the bipartite K/P-S network where K/P were randomly shuffled, and MTOR_HUMAN as a target. For each setting, three independent optimizations were run for 50 generations, with parameters sizeP=0, direct interactions=all, *n*=5,000, tolerance=15%, and cap=20.

*mTOR inhibitors analysis*. The analysis for the first data set (Figs 4 and 7, left) is sidentical as the one in option (i) of the ‘Target discrimination and random networks' section above. The functional annotation of sites was extracted from Uniprot. For sites where both GSK3B and CDK2 were possible kinases, the kinases were prioritized based on the sign of the perturbation (the perturbation on GSK3B being an activatory one, sites showing an increase were more likely to be placed downstream of GSK3B than CDK2, whose known substrate signs showed decreases in phosphorylation, consistent with an inactivatory perturbation). For the second data set (Fig. 6, right), the settings are identical but the data used to build and train the background network are those associated with the two mTOR inhibitors in Supplementary Table 4. All network visualization is done in Cytoscape.

## Additional information

**How to cite this article**: Terfve, C. D. A. *et al.* Large-scale models of signal propagation in human cells derived from discovery phosphoproteomic data. *Nat. Commun.* 6:8033 doi: 10.1038/ncomms9033 (2015).

## Supplementary Material

Supplementary Information Supplementary Figures 1-17, Supplementary Tables 1-7, Supplementary Notes 1-3 and Supplementary References
