# Supplementary material for: Large-scale models of signal propagation in human cells derived from discovery phosphoproteomic data
Source: Nat Commun. 2015 Sep 10;6:8033. doi: 10.1038/ncomms9033 (PMC4579397; doi:10.1038/ncomms9033)
Supplement: Supplementary Information — Supplementary Figures 1-17, Supplementary Tables 1-7, Supplementary Notes 1-3 and Supplementary References [file ncomms9033-s1.pdf]

Supplementary information

Supplementary Figures

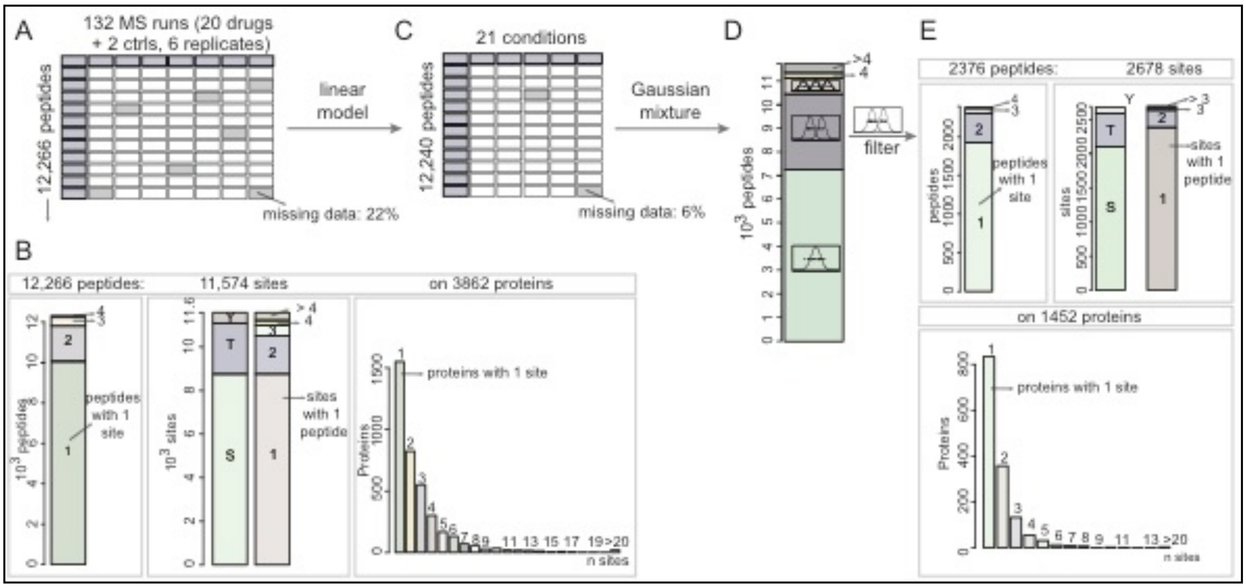

Supplementary Figure 1. Description of the data used. A. The raw data matrix contains measurements for 12,266 peptides across 132 MS runs. **B.** Most of these 12,266 peptides contain a single unambiguously localised site (10,044 peptides), while some contain multiple possible sites (1749/424/49 peptides respectively contain 2/3/4 possible sites). The 12,266 peptides contain 11,574 different phosphorylation sites, of which 75.7% are serines (S), 20.1% threonines (T) and 4.2% tyrosines (Y). The majority of these sites (77.2%) are measured on a single peptide, while some are measured on multiple peptides (respectively 15.3/4.4/1.9/1.3% of sites are measured on 2/3/4/5-14 peptides). These 11,574 sites map to 3862 proteins, most of which contain 1 (40.2%), 2 (21.4%) or 3 (14.3%) sites. **C.** After linear modelling to estimate the effect of each drug treatment, 26 peptides were lost because they didn't have enough measurements to get an estimate in any condition. The measurements for the remaining 12,240 peptides were summarised into a single estimate (with significance) for each condition. **D.** These were subjected to Gaussian mixture modelling, and a model was fitted for 11,654 peptides. Of these peptides, the majority was best fitted with a single distribution (62.3%), indicating no perturbed state in any drug treatment. Of those peptides showing a different level upon some perturbation, the majority (72.5%) are best captured with 2 components, indicating a single perturbed state. These can be used for Boolean modelling. **E.** After filtering for missing data and distribution overlap, 2376 peptides are kept, of which most contain a single unambiguously located site (80.9%). The remaining 15.8/2.9/0.3% of peptides contain 2/3/4 possible sites, respectively. These 2376 peptides map to 2678 sites, with S=78.3%, T=18.2% Y= 3.5%. Most sites are measured on 1 peptide (88.7%), while some (9.4%/1.2%/0.7%) are measured on 2/3/4- 8 peptides, respectively. The 2678 sites map to 1452 proteins, most of which contain a single site (57.4%), 2 sites (24.7%) or 3 sites (9.2%).

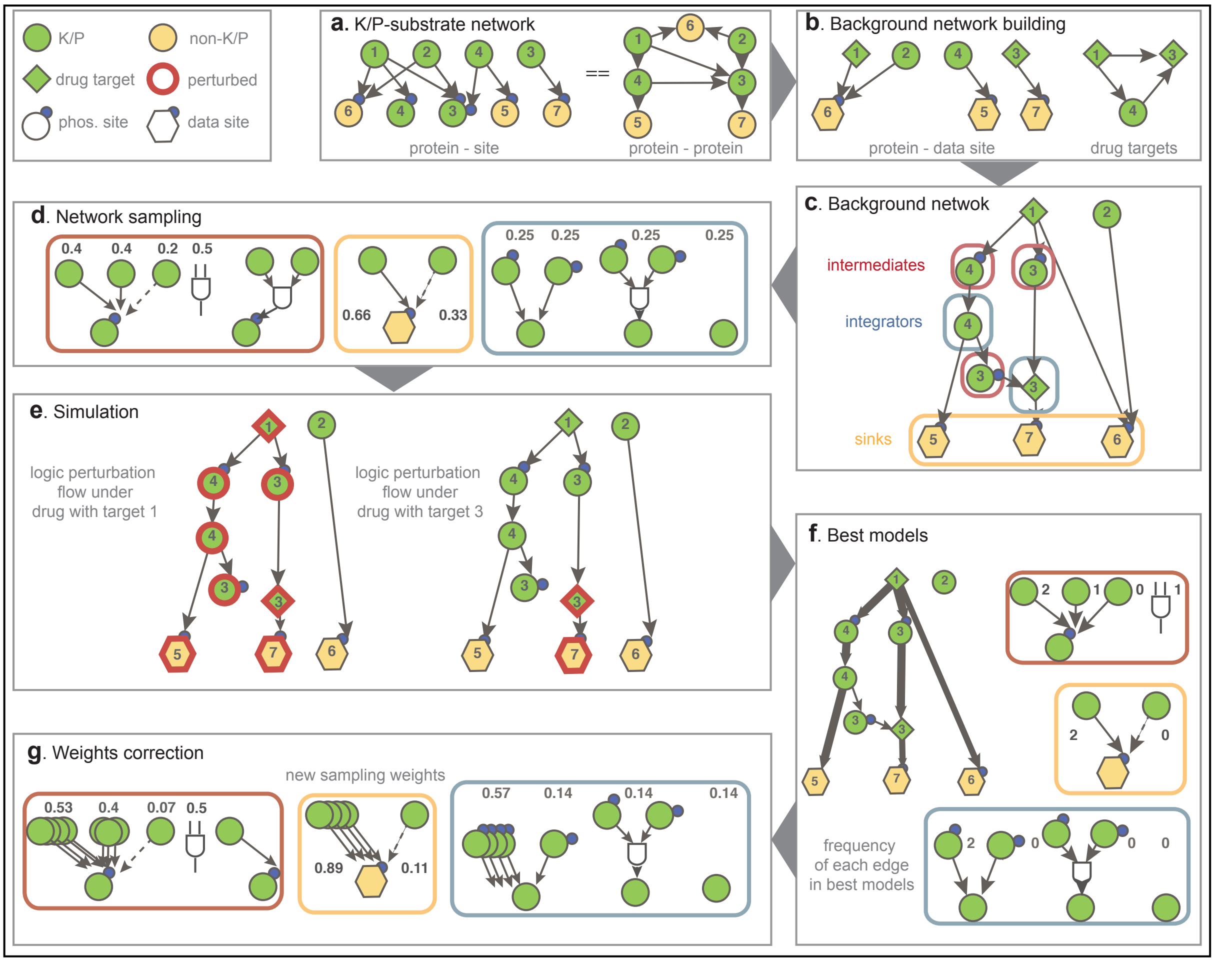

Supplementary Figure 2. Building and training a background network. A. Bipartite K/P-S network. A background network is built, for each dataset analysed (i.e. data=hexagons and drug targets=diamond nodes), from a bipartite network of K/P (green nodes)-target sites (blue dots), and its protein level equivalent. **B. Background network building.** The network reflects two main goals: i) explaining how the perturbation reached the perturbed sites, and ii) find how the different drug targets relate to each other. Therefore, we extract i) all K/P-data sites interactions and ii) a network connecting our drug targets (at the protein-protein level), then connect the two by translating protein-protein interactions back into their site-specific equivalent, and adding integrator nodes to propagate information from a kinase site to the kinase itself. **C. Background network.** The network contains three types of nodes: sinks (terminal, data nodes), integrators (protein K/P nodes that integrate regulation from sites on the K/P), and intermediates (all others). **D. Network sampling and model building.** Incoming edges for each node are sampled independently. For intermediates (red box): all possible logic combinations of input edges are allowed by i) drawing for the edges (each edge has an equal probability 1/#edges of being included, halved for predicted edges=dashed edges), ii) sampling the AND/OR gate to combine the edges (initially with equal probability); in this example, each edge has a 0.5 sampling probability such that both will be sampled simultaneously in an expected 25% of cases, neither will be sampled in an expected 25%, and either one or the other will be sampled in half of the remaining 50% of cases. Of those 25% of networks were both edges are sampled, half are expected to be combined with an AND gate (i.e. 12.5% of the total number of sampled networks). For sinks (yellow box): one edge is sampled (equal probability, halved for predicted edges), in this example [edge1, edge2] each get one of two bins, i.e. a probability of 0.5. For integrators (blue box): sample one, none or all edges (with an ‘AND’), with equal probability, e.g. this two incoming edges case has four input combinations: [edge1, edge2, edge1 +edge2, no edge], which each get one of four bins, i.e. a probability of 0.25.. **E. Simulation.** Model simulation is done by deterministically propagating

the perturbation (red contour nodes) from the drug targets, until steady state. All edges are positive because we model perturbation flow, as evidenced by increases/decreases in phosphorylation, and not these signed changes themselves, and there are only two types of gates, OR/AND (i.e. the target is set to 1 if any/all of the input nodes is/are equal to 1). **F. Scoring and generation best models.** Models are scored based on the scores S_{ij} for nodes predicted as perturbed or not perturbed, under the set of conditions analysed: $S_{model}=\Sigma_{nodes\ predicted\ P}\ S_{ij} - \Sigma_{DP\ nodes\ predicted\ C}\ S_{i,j}$ (i.e. true positive predictions (negative S_{ij}) + false positive predictions (positive S_{ij}) - false negative predictions (negative S_{ij})). The frequency of each edge in a subset of the best scoring models (represented here as the weight of edges in the background structure) is computed (numbers next to the example structures, e.g. for the intermediate node represented (red box), one of the two best models selected has the 2 leftmost edges combined with an ‘AND’ gate, and one has the leftmost gate only; for the sink example (yellow box) both best models have the left edge; for the integrator (blue box) both models have the “all edges’” option). **G. Weights correction.** Edges are “copied” in proportion of their frequency in the best models (with a maximum number called “cap”), increasing their sampling probability, i.e. information from the boxes at step d is combined with information from the boxes at step f. For intermediates (red boxes), the ’AND’ sampling probability is the average of its latest value and its frequency in the latest best models. For the integrator example (blue box), if cap=2 the new possibilities are: [edge1 x 2, edge2, (edge1 + edge2) x 2, no edge], with respective probabilities [2/6 , 1/6 , 2/6 , 1/6] (edge1: 1 bin from step d + (50%*2=1) bin from step f; edge2: 1 bin from step d; AND with both edges: 1 bin from step d + (50%*2=1) bin from step f; no edge: 1 bin from step d). For the sink example (yellow box), the new possible inputs are: [edge1 x 3, edge2] with probabilities [0.75, 0.25] (edge1: 1 bin from step d + (100%*2=2) bins from step f; edge2: 1 bin from step d). For the intermediate example above, edge1and edge2 will be sampled with respective probabilities 3/5 and 2/5 (edge1: 1 bin from step d + (100%*2=2) bins from step f; edge2: 1 bin from step d + (50%*2=2) bins from step f), and the AND will be sampled with probability (0.5+0.5)/2 (average of the sampling weight in box d and the frequency in best models from box f). The numbers above each input represent the new sampling probabilities at the second generation, matching to step d (first generation).

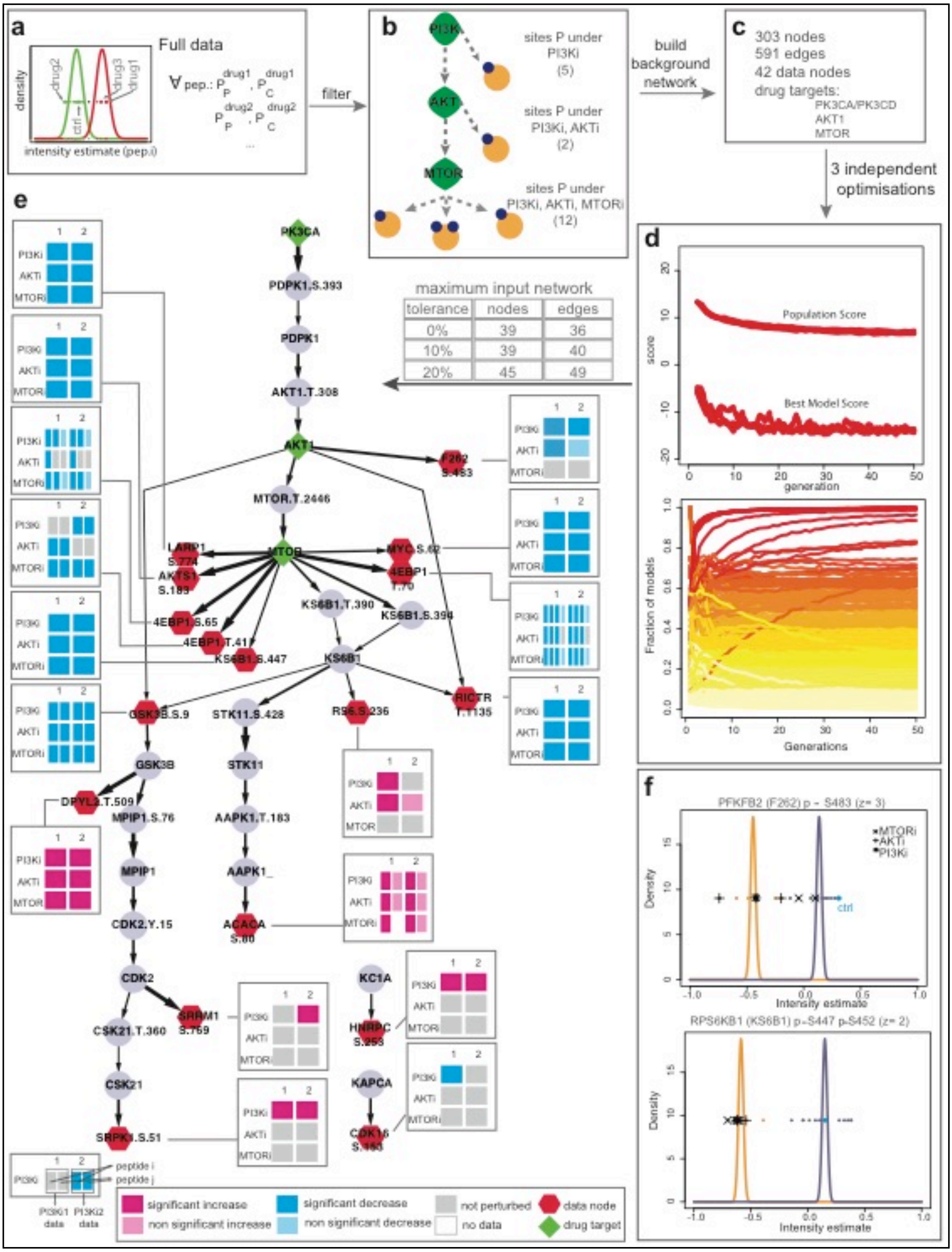

Supplementary Figure 3. Proof of principle on reduced scope data. A,B. Sites with a perturbation pattern consistent with the canonical PI3K-AKT1-MTOR hierarchy are selected. **C.** The network built from these sites and drug targets is used for three independent optimisations. **D.** The three optimisations converged to similar scores (top panel) and stable averaged edge frequencies (lower panel). **E.** Averaged frequencies are used to extract a network of most likely paths from drug targets (green diamonds) to data (red hexagons) with various levels of

tolerance around the highest frequency edge (10% represented). Panels represent the data for a given site under each of our three pairs of kinase inhibitors; subdivided panels= multiple peptides mapping to the site. **F.** Example data and GMMs used to compute the scores (blue=control distribution, yellow=perturbed distribution). Note: all node names are Uniprot identifiers (without the _HUMAN suffix).

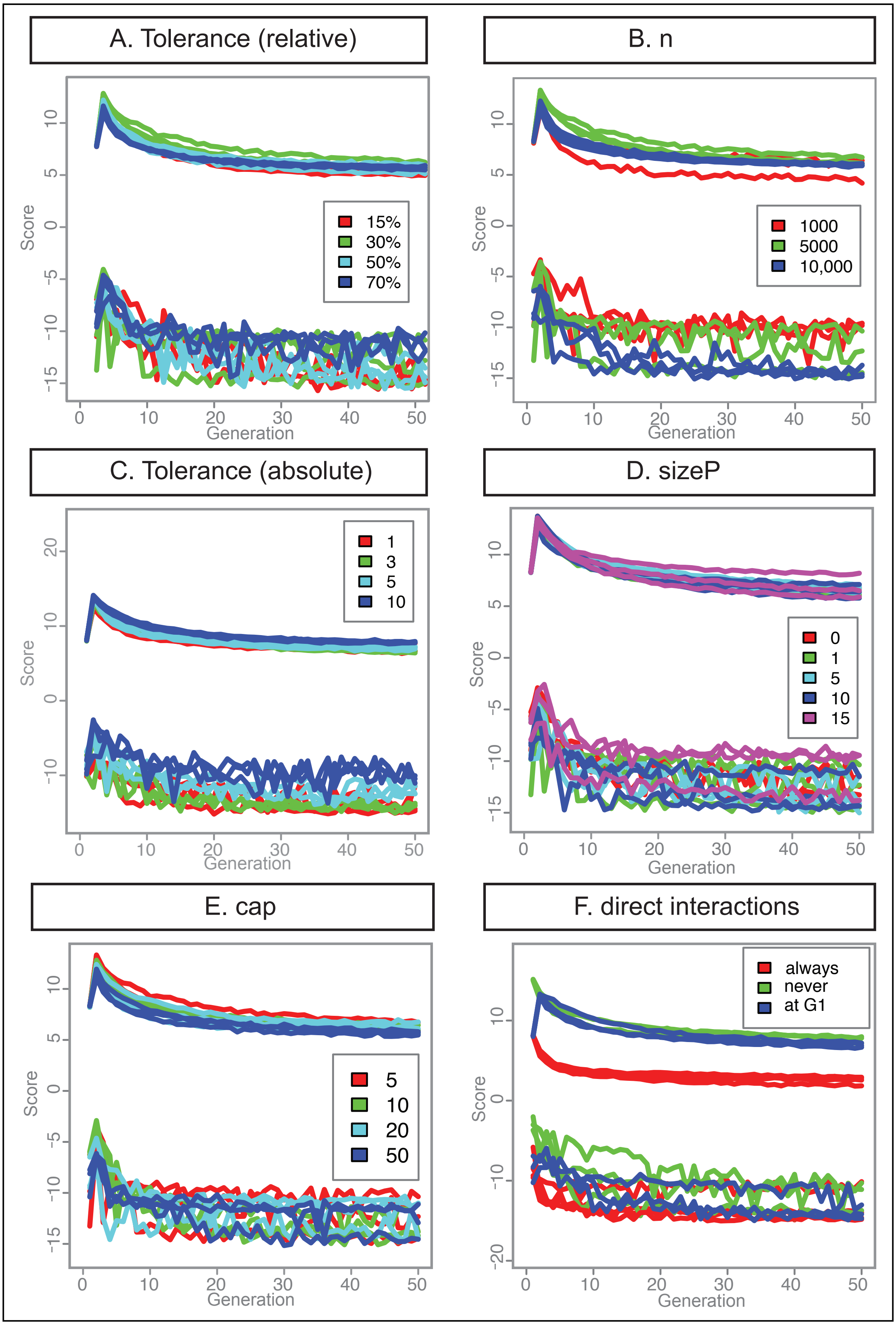

Supplementary Figure 4. Influence of training parameters. Population (top) and best model (bottom) scores are plotted for 50 training generations. Three independent optimisations of the reduced scope data presented in Fig. 3. are performed for each set of

parameters tested. G1=first generation of optimisation. Scores are shown without any size penalty because we found model size to be almost identical under any combination of parameters, due to the way that the sampling works. Unless stated otherwise, all optimisations are performed with settings direct interactions=G1, cap=5, n=5000, tolerance=30%, sizeP=1. **A.** The relative tolerance is the window of scores around the best score of each generation that is used to select the best models (in % of the best score). This parameter seems to have little influence on the scores between 15 and 50%, performing worse at the best model level at 70%. **B.** n is the number of models sampled at each generation. Unsurprisingly, higher n leads to better best models. In terms of average population scores however, most of the curves stabilise around the same area, with the lower numbers of models being more prone to outliers on either side (i.e. better or worse scores). In practice we found 500 to be a good compromise between optimisation time and stability for most models tested. **C.** The absolute tolerance is the same as the relative tolerance except that the window is fixed throughout generations. There is here a clear gradient from better (low tolerances) to worse (high tolerances). Based on this much higher sensitivity and difficulty to set a score range a priori, we would suggest to use relative tolerances. **D.** The size penalty is a factor that, multiplied by the fraction of edges sampled in each model, can be added to the model scores to account for model size in the optimisation process. At the population average level, the higher the size penalty, the bigger the variability between independent optimisations. In terms of best model scores, the highest size penalty shows poor performance. Size penalties have close to no influence on the size of models (due to the nature of the sampling process, most nodes will pick an incoming edge, so it is not so much the number of edges as their identity that varies from one model to another). However, higher size penalties allow poorer models with slight differences in size to sometimes reach better scores, resulting in a bigger variability between optimisations. We therefore excluded the size term in all optimisations. **E.** The cap is the maximum number of times that an edge is “copied through” in the background network, at the end of a generation (see Supplementary Fig. 2). This parameter determines the speed of convergence by influencing how much of an advantage edges in best models have at the next generation. The higher the cap, the better the average population score. The best model scores however seem to be largely unaffected. **F.** The “direct interactions” setting refers to the possibility to force include the direct (non-predicted) interactions between sites perturbed and drug targets at all generations, the first generation or not at all (in order to simplify the training process by forcing obvious interactions in or giving them an advantage in starting weights). Clearly, including the direct interactions at all generations produces better scores and allows the optimisation to focus on capturing other sites.

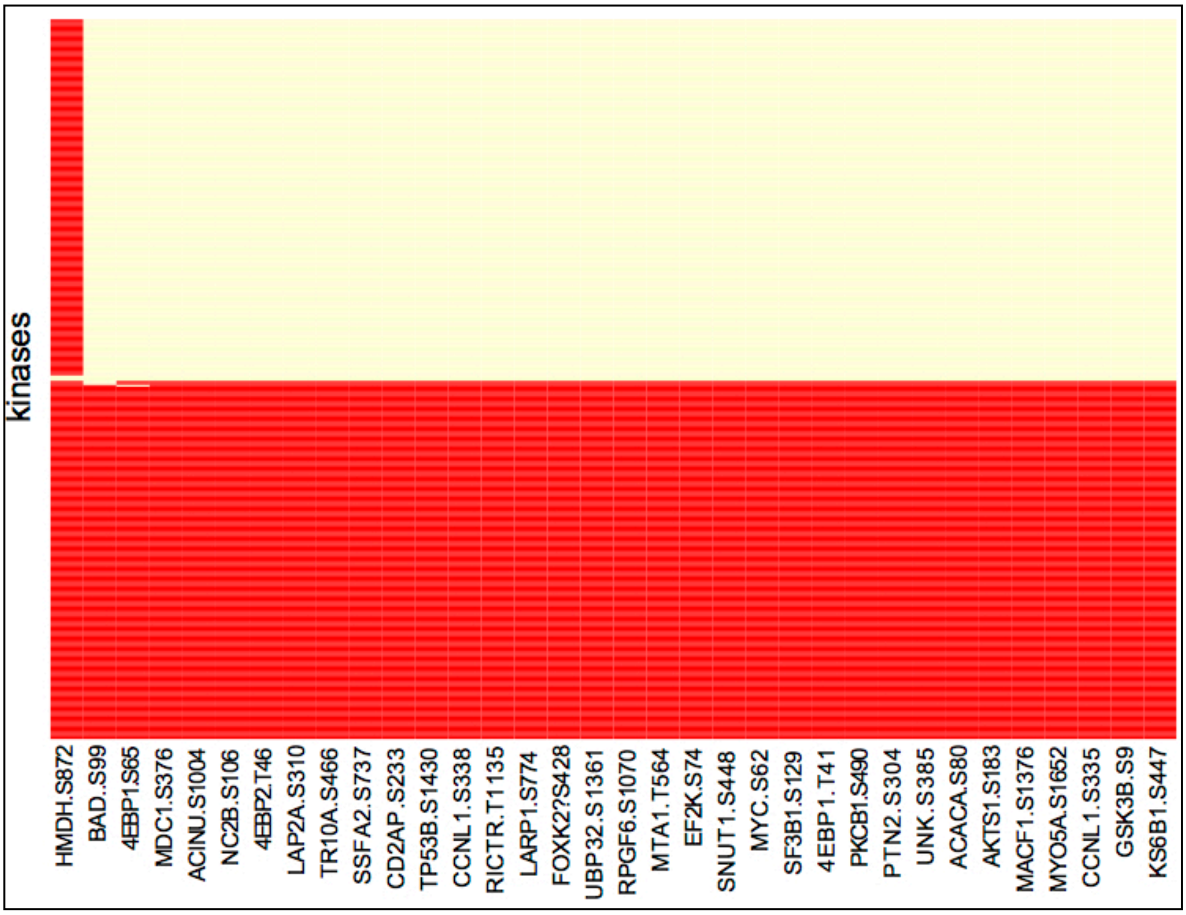

Supplementary Figure 5. Target discrimination and information content in the network: connecting the MTORi sites. For each site perturbed under MTOR inhibition (columns) and each K/P in the K/P-substrates network, we represent a yellow square if the K/P has a path to the site (in the full network of K/P-S interactions), and a red square otherwise. With the exception of HMDH.S.872, which is only reached by three K/P, all sites can be reached by the same enzymes, which represent over half of the candidates K/P. This implies that looking exclusively at the existence of a path to determine which kinases are potential drug targets is likely to be uninformative.

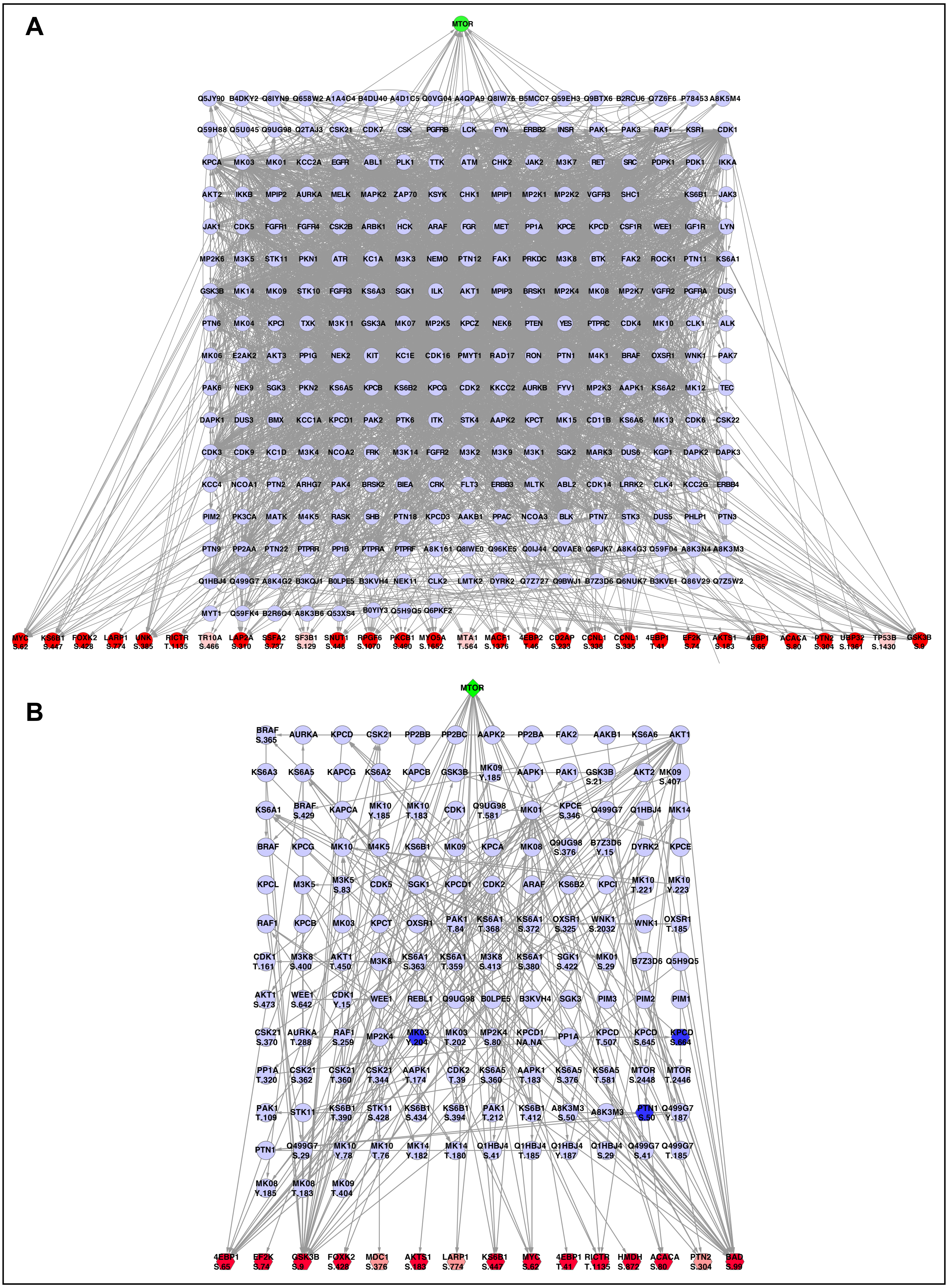

Supplementary Figure 6. MTOR inhibitors data: full background. This represents the full network of possible paths between MTOR and the sites found to be perturbed under MTOR

inhibition (A including, and B excluding predicted edges). A. All paths are represented at the protein level, for clarity (apart from the step between perturbed sites and all their known potential kinases). In consequence, every edge can represent multiple underlying edges, one for each site on the target K/P that is phosphorylated/dephosphorylated by the source K/P. Clearly, without the optimisation steps in *PHONEMeS*, this knowledge is of very limited use to interpret the data. B. Excluding predicted interactions, the network becomes easier to manipulate (261 edges and 163 nodes) but can only capture 15 of the 32 sites perturbed under MTOR inhibition. To capture the remaining 17 sites in our data, in the absence of a method like *PHONEMeS* that can pick out the kinases likely to lie on perturbation paths and connect predicted sites to these kinases, the only option available is to use the full (hardly visualisable) background in A.

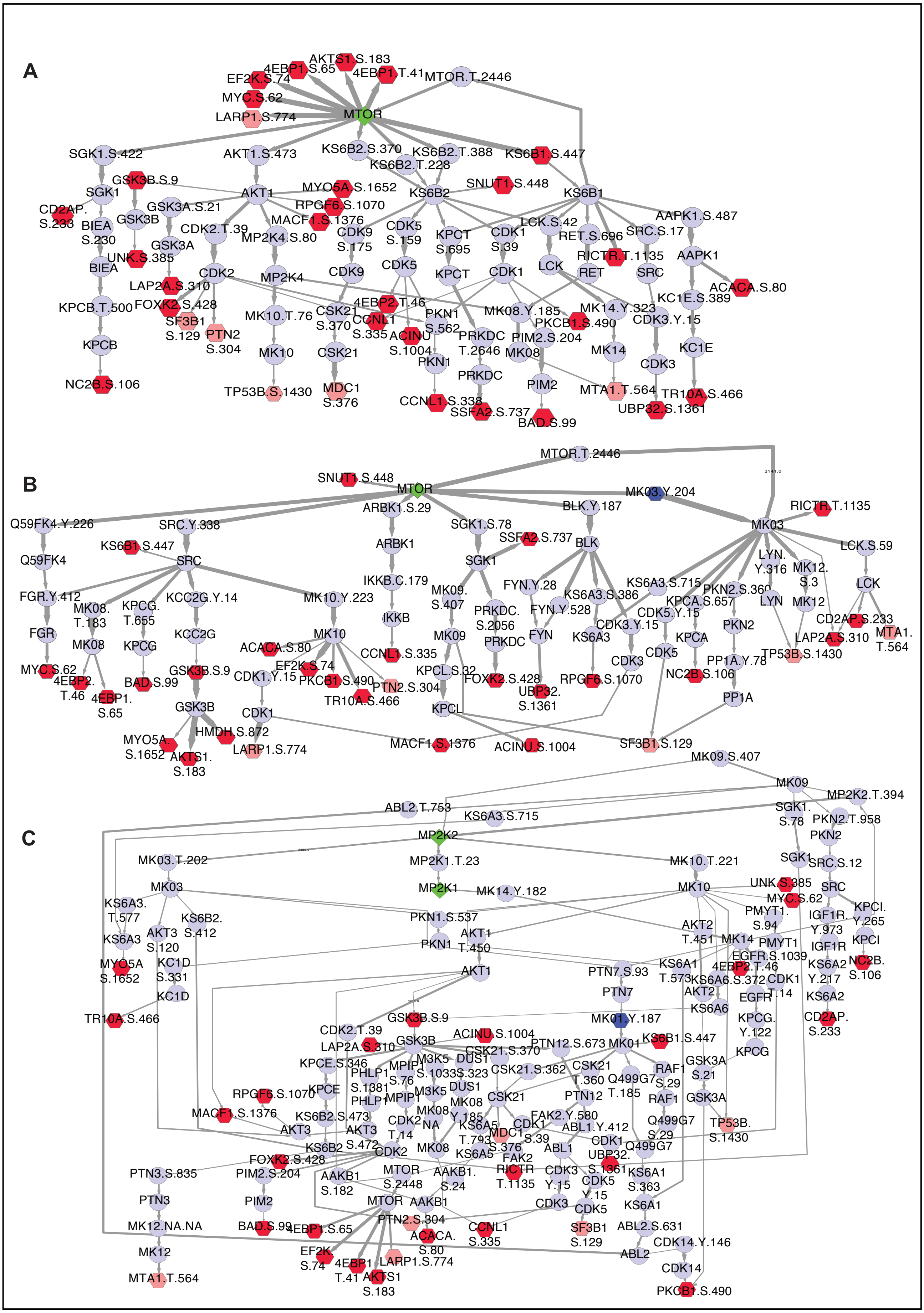
A
B
C

Supplementary Figure 7. Target prediction and network information content. Networks resulting from the analysis on fig.4 (corresponding to the simplified representations on fig.4C), all with 20% tolerance. **A.** Real network/correct target (33 out of 34 perturbed nodes connected to MTOR, average shortest path length of 4.3; 92 nodes in total). **B.** Resulting network for one of the three randomised networks/real target (30 out of 34 perturbed nodes connected to MTOR, average shortest path length of 4.9; 83 nodes in total). **C.** Real network/incorrect target (30 out of 34 perturbed nodes connected to MP2K1/MP2K2, average shortest path length of 8.1; 141 nodes in total). Most sites are connected to the designated targets in all cases but the paths are shorter with the correct target than with the incorrect ones. The random networks show a major difference in terms of number of sites explained directly by the drug target or its first neighbours (14 sites for the real network *vs.* 4 sites for this random network).

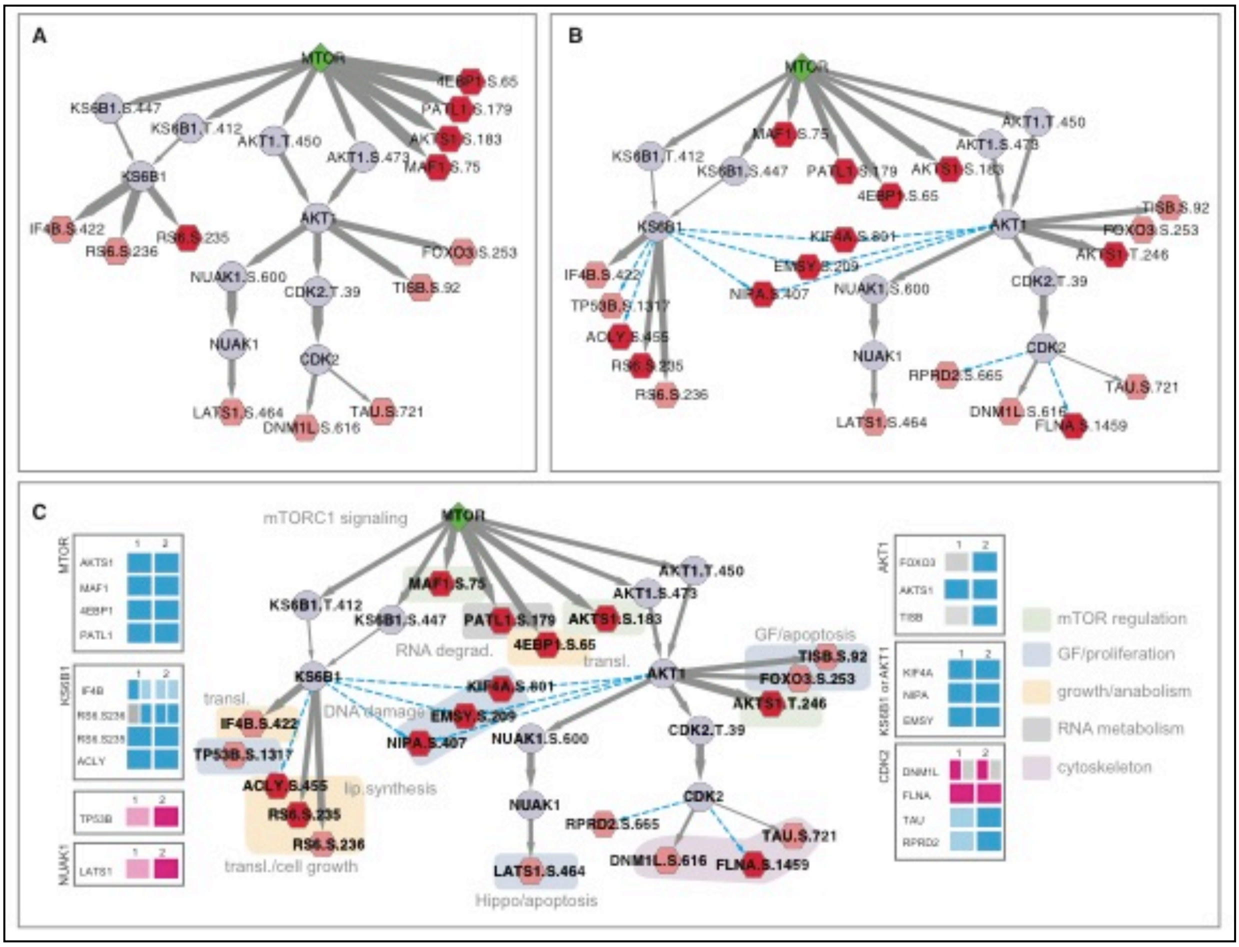

Supplementary Figure 8. Modelling the effect of MTOR inhibition in a publicly available data set[13]. A. 12 sites perturbed under one of the two MTOR inhibitors in the validation data were connected to MTOR in six independent optimisations, using exclusively experimentally demonstrated interactions. Results of these were combined into average frequencies in final populations of models, which were used to extract a best consensus model with 5% tolerance around the highest frequency edges. This model recapitulates effects on known substrates of MTOR, and shows a good agreement with the data obtained with a different mass spectrometer and reagents in Fig. 4. This network is less constrained and shows a lower agreement between the two drugs when looking at sites further downstream from MTOR. This could be due to the lower coverage, and possible lower quality of this data set (due partially to the type of spectrometer used, and to the data processing approach, see *Wilkes et al.*[22]). B. Sites that were found to be perturbed but did not have an experimentally demonstrated kinase were connected to the optimised network based on predictions from *networKIN*[17]. Note that sites which could be connected to a kinase that is a first neighbour of mTOR are more likely to be consistently identified under both drug treatments, further supporting our network-based prioritisation of potential kinases. C. Predictions were prioritised based on the sign of the perturbations observed, and functional annotations of proteins were extracted from *Uniprot*. We can see that many of the functional themes and network connections that were identified with the main data in Fig. 4 are also found here, even though the two sets of experiments involved different drugs, spectrometric equipments and as a consequence vastly different data sets and perturbed peptides. Additionally, this analysis shows that even with data with lower coverage and less advanced technology it is possible to obtain a network that is informative both in terms of the functional effect of a drug treatment and in terms of the prioritisation of kinase-substrate relationships downstream of this drug treatment.

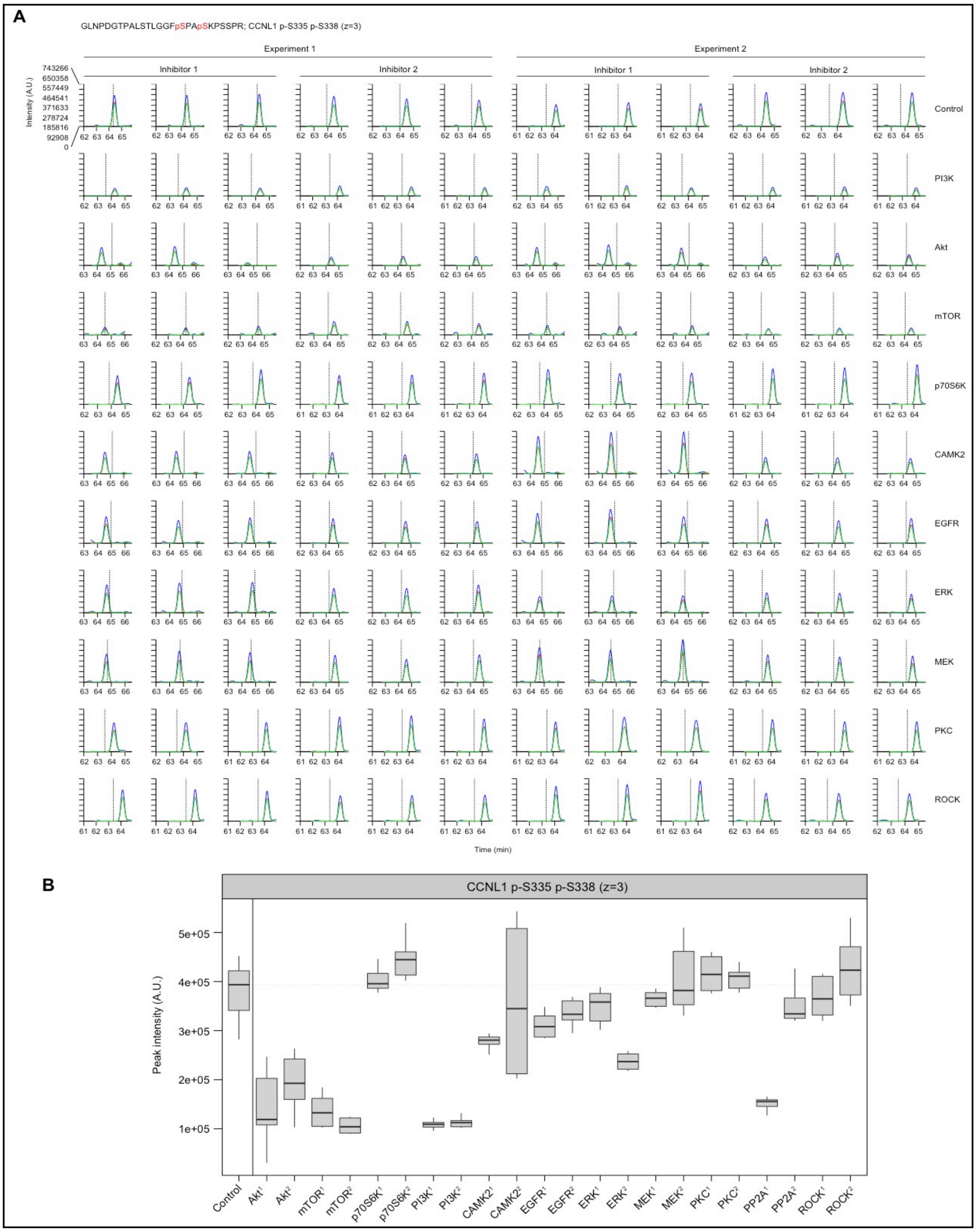

Supplementary Figure 9. Inhibition of mTORC1/2 or Akt decreases phosphorylation of Cyclin-L1. A. Extracted ion chromatograms for the ion representing two phosphorylation sites on Cyclin-L1 (CCNL1) under each of the inhibitor treatments (blue lines, first isotope; red lines, second isotope; green lines, third isotope). B. Box plots summarising the raw intensity data shown in (B). Dotted line represents the median intensity under treatment with DMSO vehicle control. This phosphorylation of this CCNL1 peptide was decreased 2 to 5 fold following Akt and mTORC1/2 inhibition, as well as PI3K inhibition, but not P70S6K, which is consistent with the network prediction in Fig. 4.

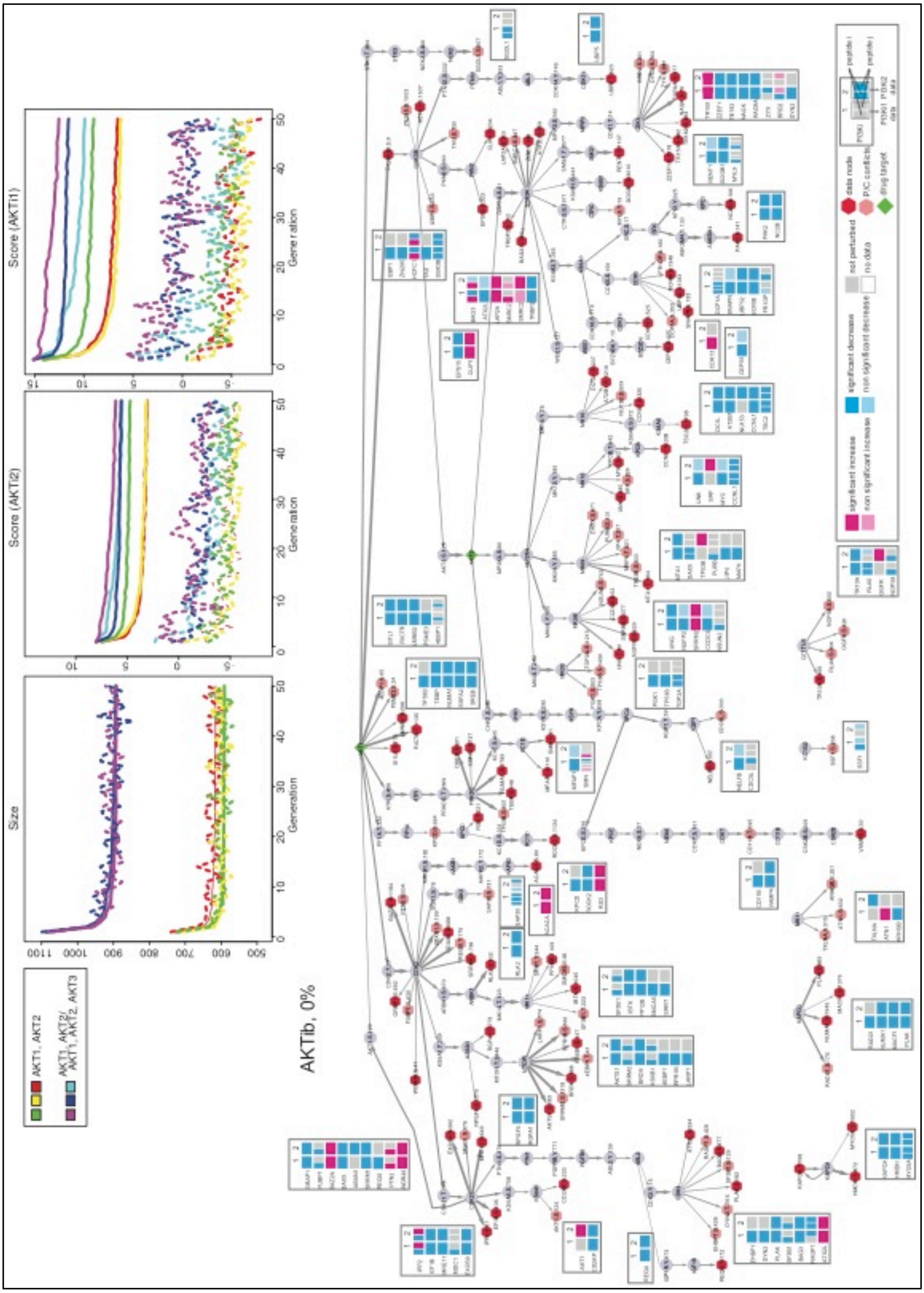

Supplementary Figure 10. Modelling the effects of the Akt inhibitors Akt Inhibitor VIII (AKTi1) and MK-2206 (AKTi2). Results of 3 rounds of optimisation with different potential targets for AKTi1 and AKTi2. Top: population average (continuous) and best model (dashed)

size (number of edges) and scores along 50 generations of optimisation when both drugs are assumed to target AKT1 and AKT2 (red, green and yellow curves, AKTib) or when AKTi2 is assumed to also target AKT3 (blue and purple curves). Bottom: network resulting from the combined output of the 3 rounds of optimisations AKTib, visualised with 0% tolerance around the highest frequency input edges. Edges widths represent the average frequency at which the edge is present in the population of networks at the last generation of the optimisation.

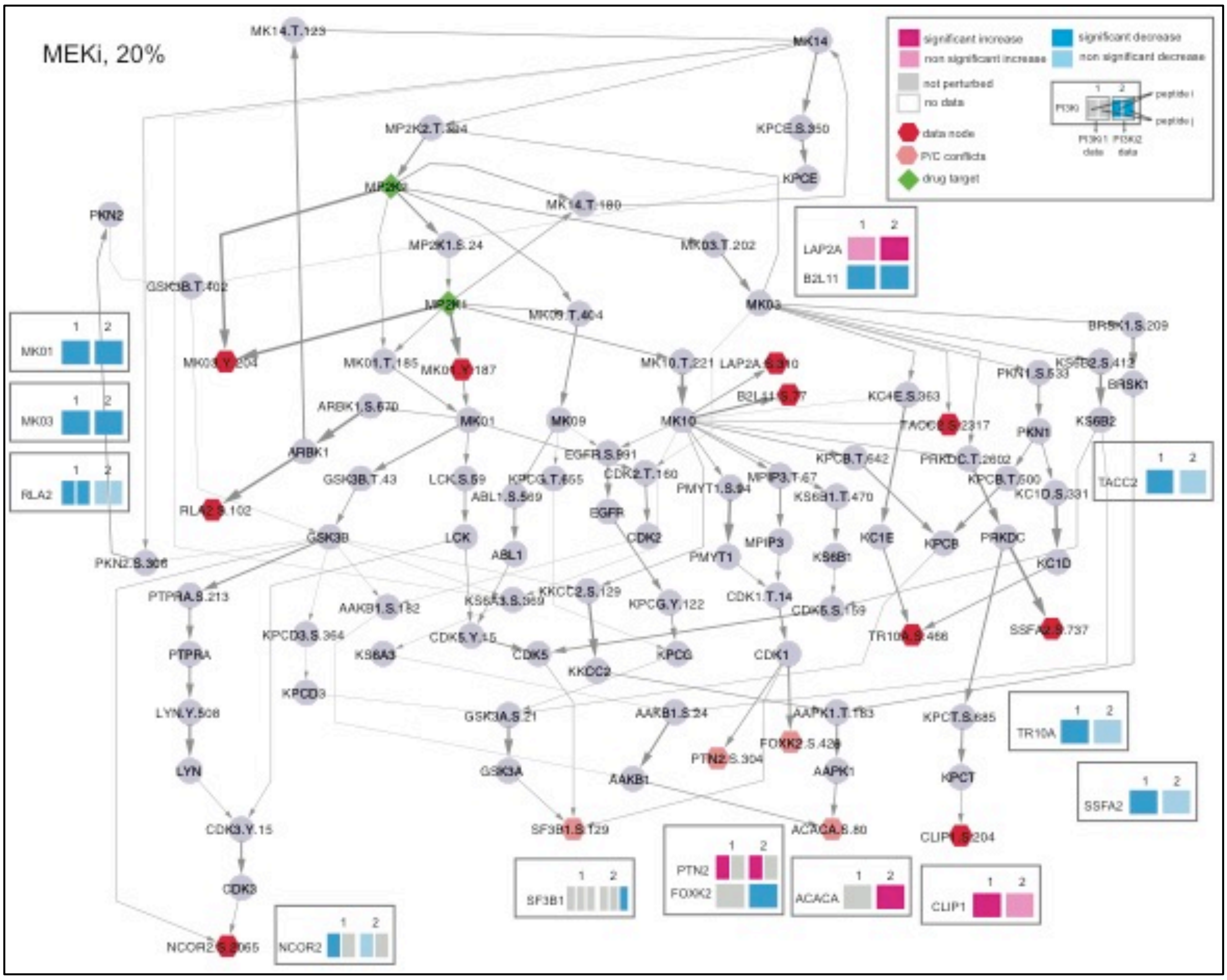

Supplementary Figure 11. Modelling the effect of the MEK inhibitors GSK-1120212 (MEKi1) and U0126 (MEKi2). Network resulting from the combined output of three rounds of optimisations with both drugs assumed to have the same targets MP2K1 and MP2K2, visualised with 20% tolerance around the highest frequency input edges. Edges widths represent the average frequency at which the edge is present in the population of networks at the last generation of the optimisation.

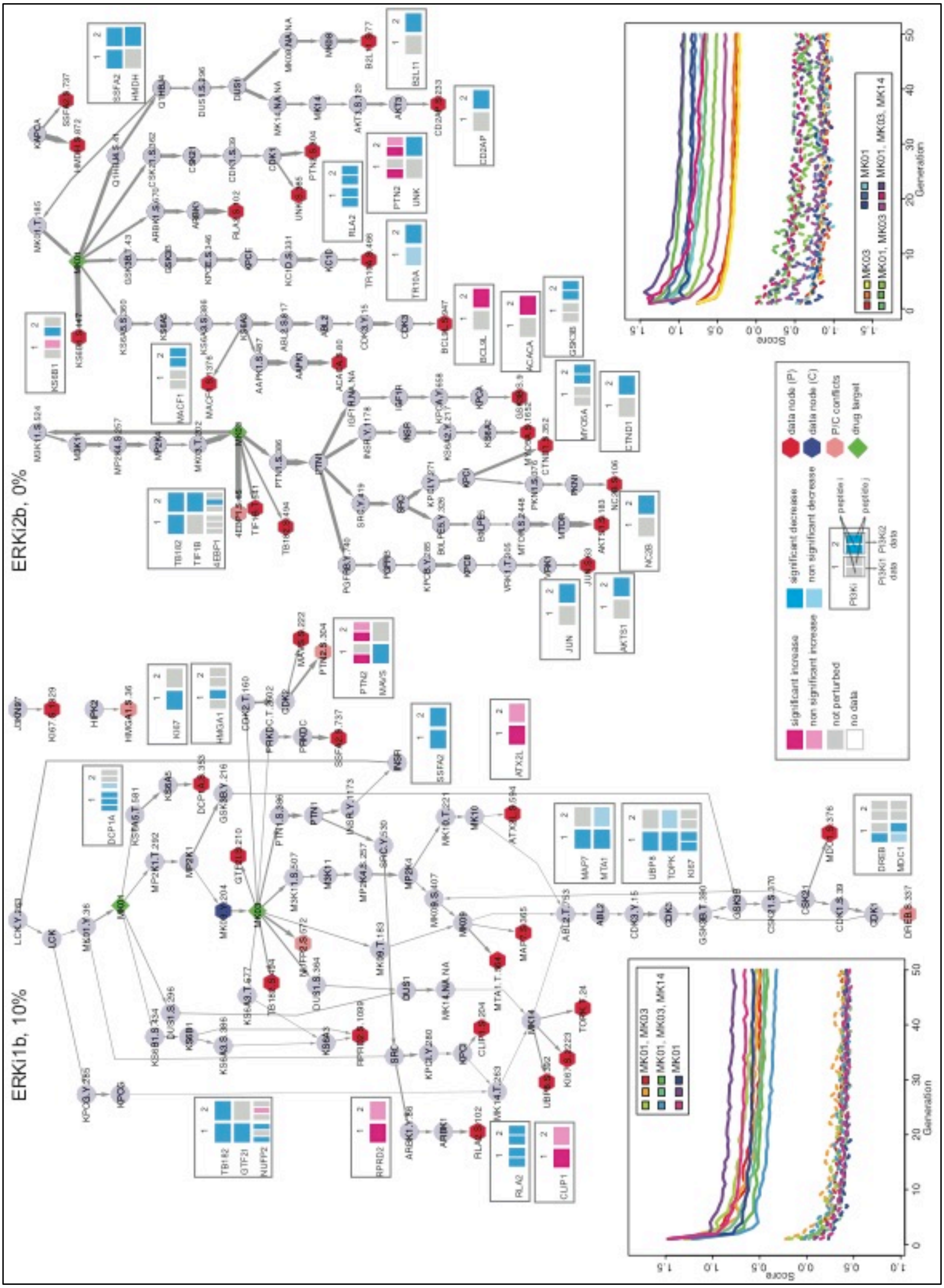
ERKi1b, 10%
ERKi2b, 0%
significant increase
non significant increase
not perturbed
no data
significant decrease
non significant decrease
data node (P)
data node (C)
P/C conflicts
drug target
Score
Generation

Supplementary Figure 12. Modelling the individual effect of the Erk inhibitors I (ERKi2) and II (ERKi2). Results of three rounds of optimisation with different potential targets for ERKi1 and ERKi2. Top: networks resulting from the combined output of the three rounds of optimisations ERKi1b (ERKi1 with targets MK01 and MK03) and ERKi2b (ERKi2 with targets MK01 and MK03), visualised with 10% and 0% tolerance around the highest frequency input edges, respectively. Edges widths represent the average frequency at which the edge is present in the population of networks at the last generation of the optimisation. Bottom: population average (continuous) and best model (dashed) scores along 50 generations of optimisation: left, when ERKi1 is assumed to target MK01 and MK03 (red, light green and orange curves), MK01, MK03 and MK14 (light blue, turquoise and dark green curves) or MK01 only (dark blue, pink and purple curves); right, when ERKi2 is assumed to target MK03 only (red, orange and yellow curves), MK01 only (blue curves), MK01 and MK03 (green curves) or MK01, MK03 and MK14 (pink and purple curves).

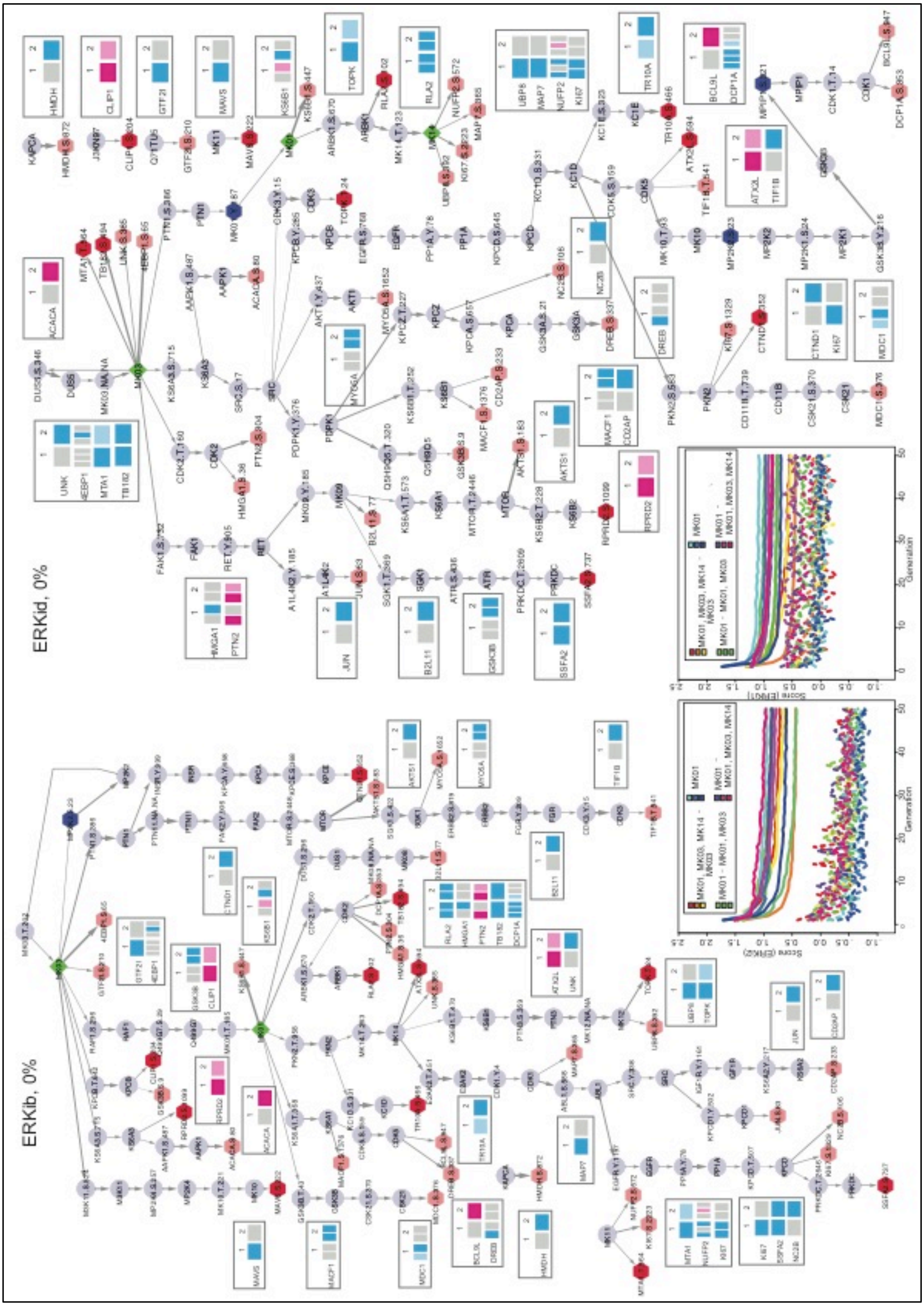

ERKib, 0%
ERKid, 0%

Supplementary Figure 13. Combined modelling of the effect of the Erk inhibitors I (ERKi2) and II (ERKi2). Results of three rounds of optimisation with different potential targets for ERKi1 and ERKi2. Top: networks resulting from the combined output of the three rounds of optimisations ERKib (both drugs with targets MK01 and MK03) and ERKid (ERKi2 with targets MK01 and ERKi1 with targets MK01, MK03 and KM14), visualised with 0% tolerance around the highest frequency input edges. Bottom: population average (continuous) and best model (dashed) scores along 50 generations of optimisation: left, for ERKi1 and right, for ERKi2, when ERKi1 and ERKi2 are assumed to i) both target MK01 (blue curves), ii) target MK01, MK03 and MK14 (ERKi1) i1) and MK01 and MK03 (ERKi2) (green curves) or iv) target MK)! (ERKand MK03 (ERKi2) (red, orange and yellow curves), iii) target MK01 (ERKi1) and MK01, MK03 and MK14 (ERKi2) (pink and purple curves). Network and data representation conventions are as in Supplementary Figs.10-12.

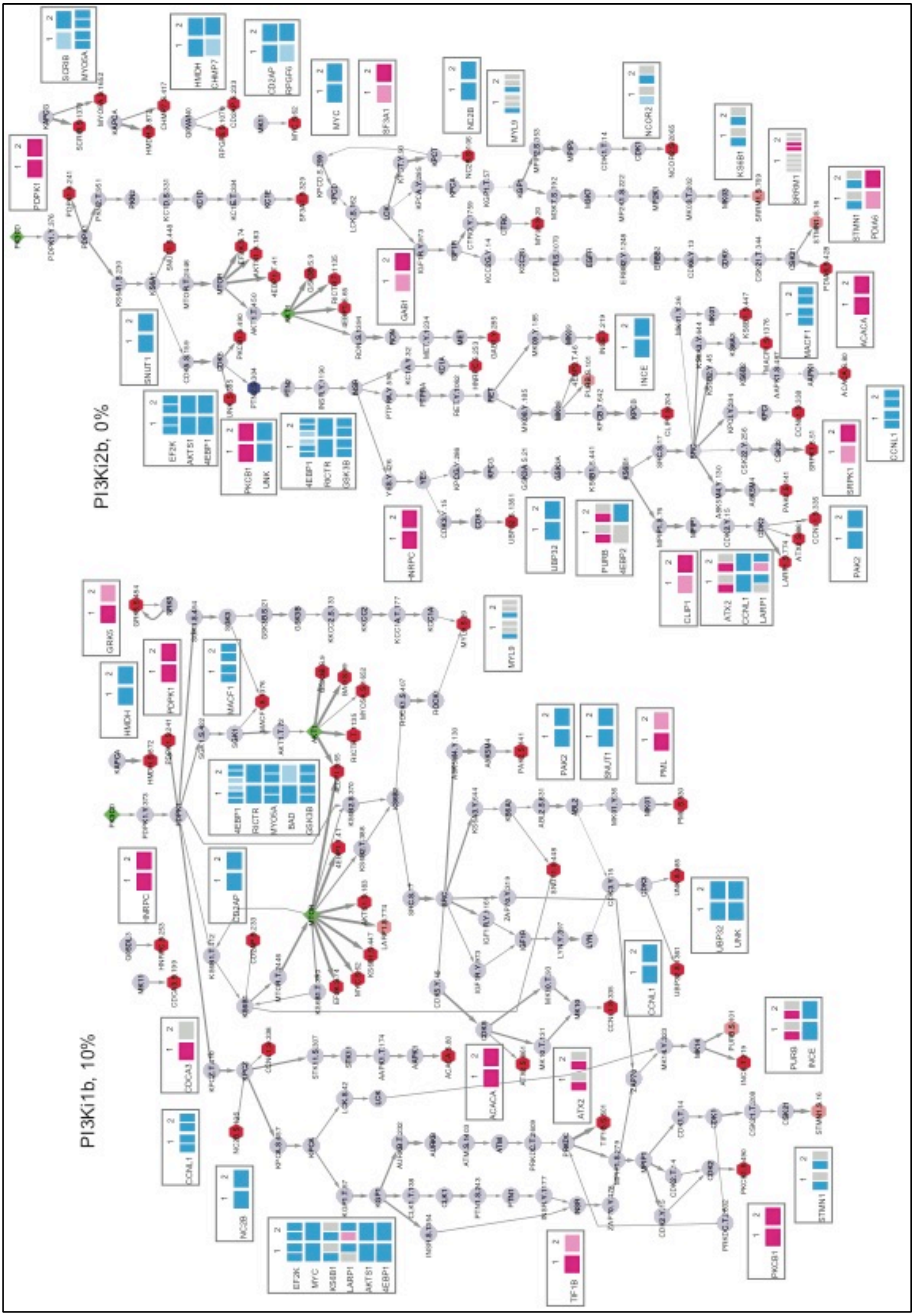
PI3Ki2b, 0%
PI3Ki1b, 10%

Supplementary Figure 14. Modelling the individual effect of the PI3K inhibitors GDC-0941 (PI3Ki1) and PI-103 (PI3Ki2). Network resulting from the combined output of three rounds of optimisations: left, for PI3Ki1, assumed to have PK3CA, PK3CD, MTOR and AKT1 as targets; and right, for PI3Ki2 assumed to have PK3CA, PK3CD and AKT1 as targets; visualised with 10% and 0% tolerance around the highest frequency input edges, respectively. Network and data representation conventions are as in Supplementary Figs.10-12.

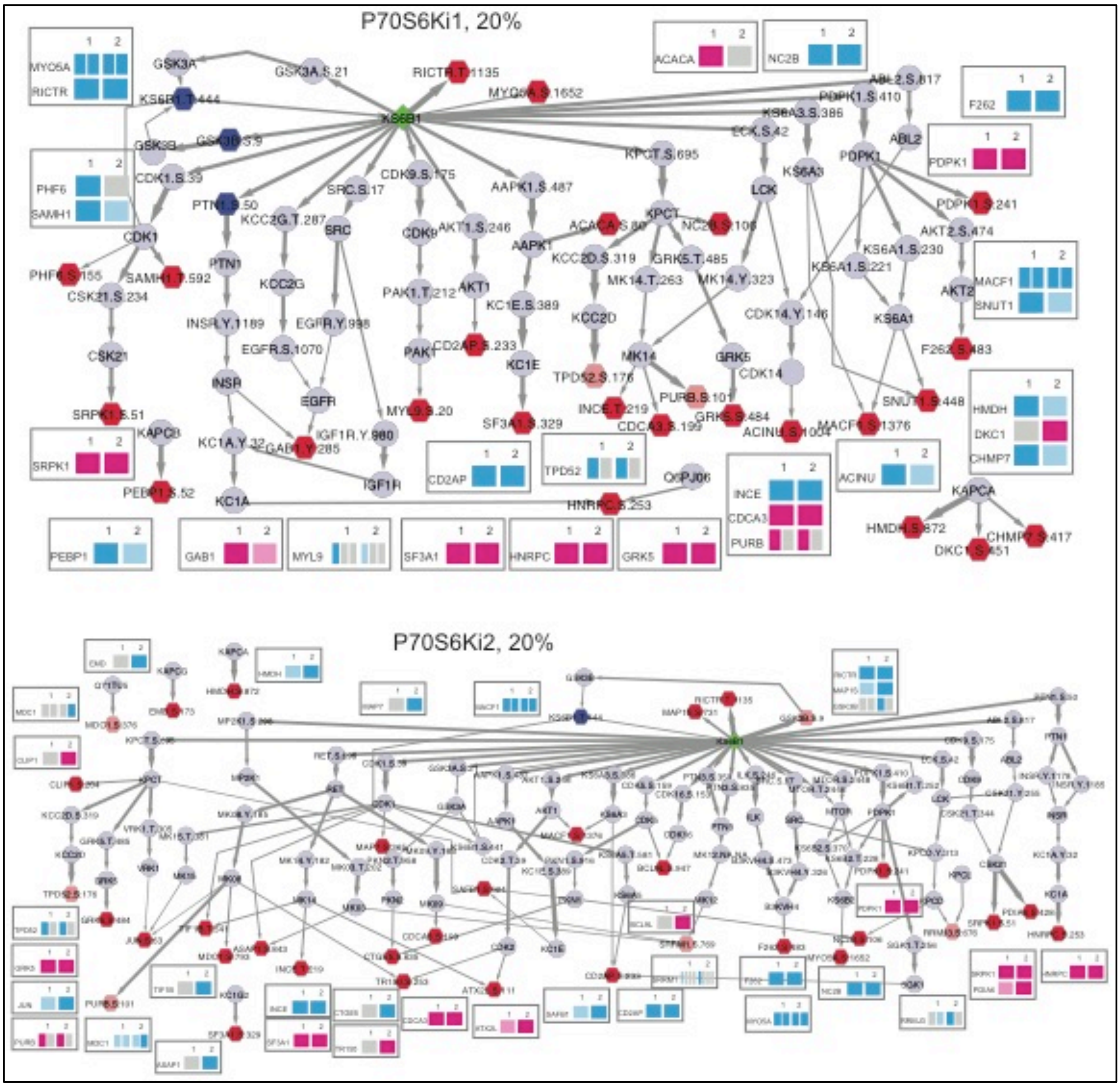

Supplementary Figure 15. Modelling the individual effect of the P70S6K inhibitors PF-4708671 (P70SKi1) and S6K Inhibitor (P70S6Ki2). Network resulting from the combined output of three rounds of optimisations: top, for P70S6Ki1, and bottom, for P70S6Ki2, both assumed to have KS6B1 as target; visualised with 20% tolerance around the highest frequency input edges. Network and data representation conventions are as in Supplementary Figs.10-12.

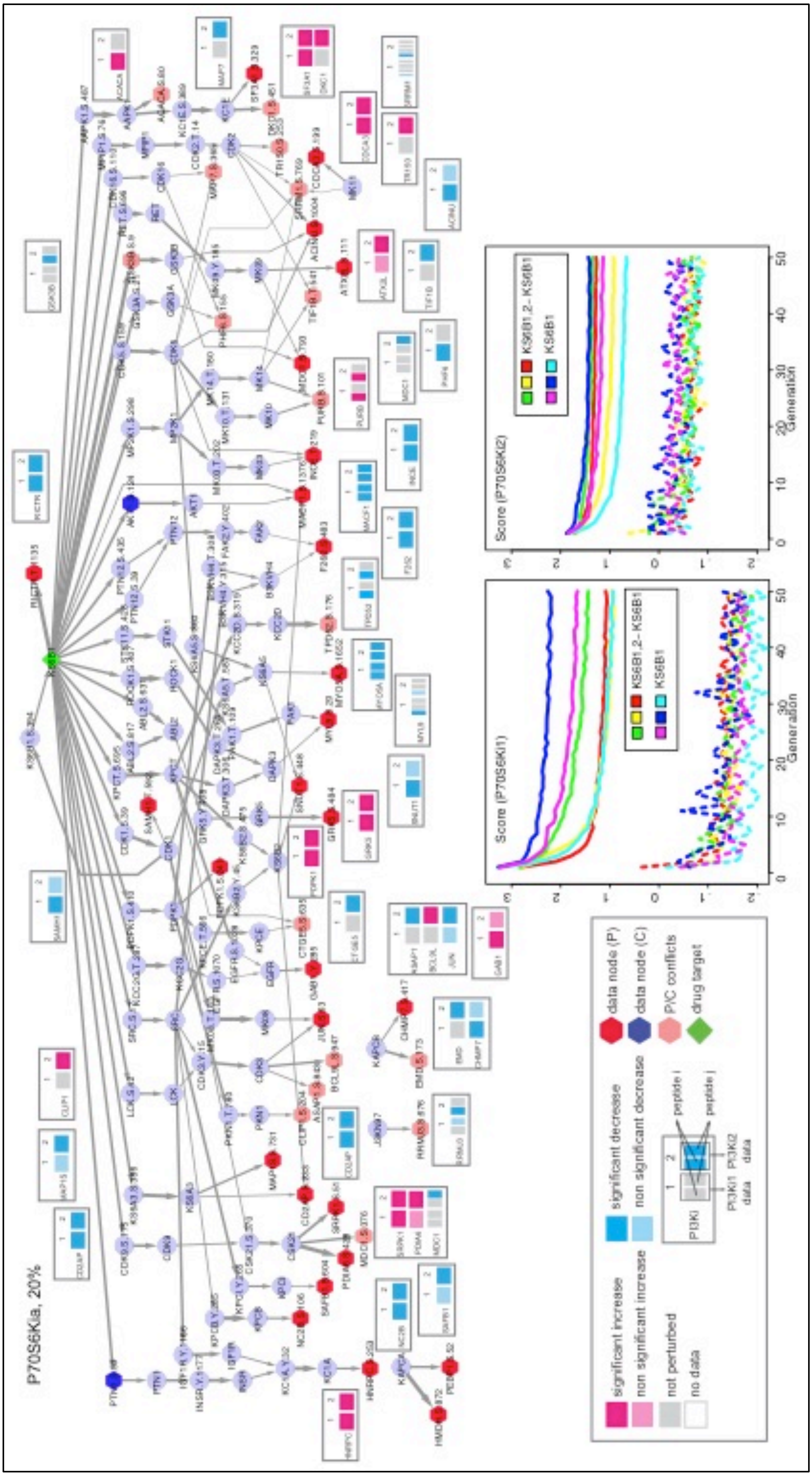

P70S6Kia, 20%
Score (P70S6Ki1)
Score (P70S6Ki2)
Generation
significant increase
non significant increase
not perturbed
no data
significant decrease
non significant decrease
data node (P)
data node (C)
P/C conflicts
drug target

Supplementary Figure 16. Combined modelling of the effect of the P70S6K inhibitors PF-4708671 (P70SKi1) and S6K Inhibitor (P70S6Ki2). Results of three rounds of optimisation with different potential targets for P70S6Ki1 and P70S6Ki2. Top: network resulting from the combined output of three rounds of optimisation in which both drugs are assumed to have KS6B1 as their only target, visualised with 20% tolerance around the highest frequency input edges. Bottom: population average (continuous) and best model (dashed) scores along 50 generations of optimisation: left, for P70S6Ki1 and right, for P70S6Ki2, when they are both assumed to have KS6B1 as their only target (pink and blue curves) or when P70S6Ki1 is assumed to additionally target KS6B2. Network and data representation conventions are as in Supplementary Figs.10-12.

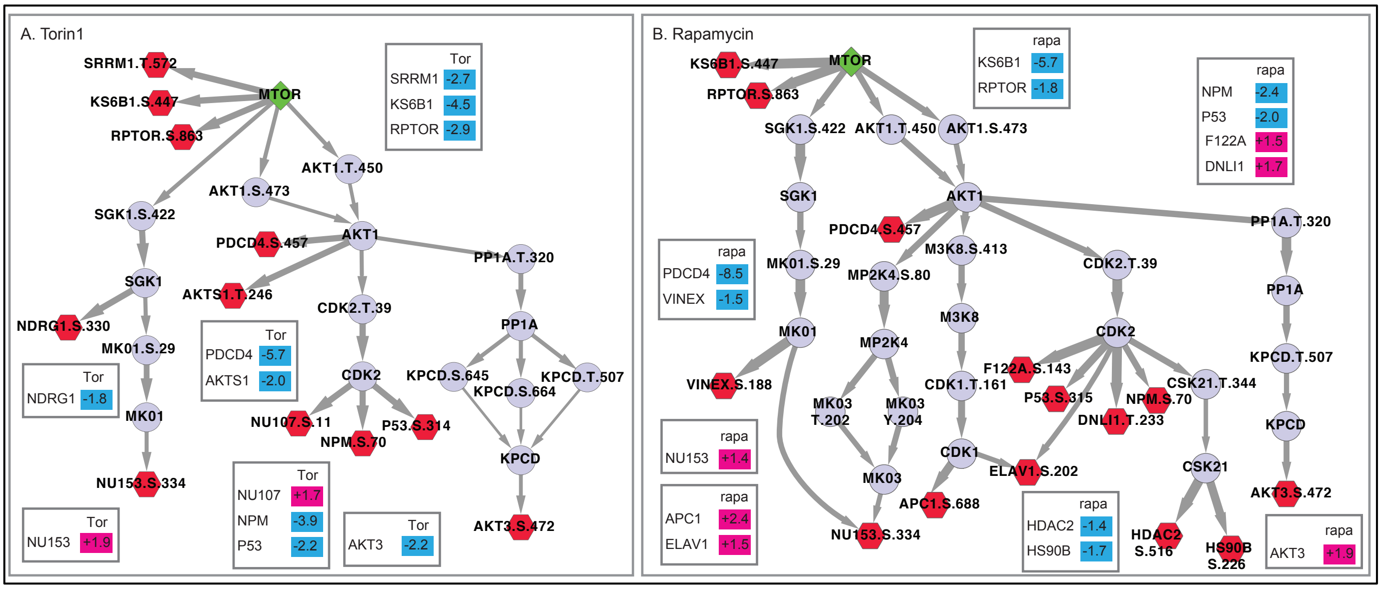

Supplementary Figure 17. Analysis of the mTOR inhibition data by Hsu et al.[13]. Published iTRAQ labelled data obtained after inhibition of mTORC1 by Rapamycin (B) and mTORC1 and mTORC2 by Torin1 (B) in HEK-293E cells (in insulin-stimulated cells) was analysed using *PHONEMeS*. The networks show the results of *PHONEMeS* analyses run separately for the two drugs, shown with 20% tolerance around the best incoming edges. The data shows the cluster assignment (pink/blue=more than 2.5MAD larger/smaller than the average $\log_2$(drug/insulin)), and the average $\log_2$(drug/insulin) for the peptide mapping to the indicated phosphorylation site (two replicates). Red nodes are sites that are perturbed by the drug treatment, grey nodes are nodes for which no information is available in the data, green nodes are nodes that are designated as drug targets. The edge widths reflect the frequency with which the edge is present in the population of models at the end of the optimisation.

Supplementary Tables

Supplementary Table 1. Drugs table. Small molecule inhibitors used to acquire the main dataset in this work (see Wilkes et al.[22]), used in Figs. 1, 3, 4, 5, 6a, 7 left side, and Supplementary Figs 1, 3, 4, 5.

Drug	Concentration (μM)	Name	chEMBL ID
AKT Inhbitor VIII	1	AKTi1	258844
MK-2206	1	AKTi2	1079175
GSK-1120212 (trametinib)	0.5	MEKi1	-
U0126	10	MEKi2	100473
KN-63	10	CAMKi1	28324
KN-62	5	CAMKi2	155333
PD-168393	1	EGFRi1	285063
PD-153035	10	EGFRi2	29197
ERK Inhibitor I	40	ERKi1	1403932
ERK Inhibitor II	30	ERKi2	259551
KU-0063794	1	MTORi1	1078983
Torin-1	1	MTORi2	1256459
GDC-0941	1	PI3Ki1	573393
PI-103	1	PI3Ki2	521851
PF-4708671	20	P70S6Ki1	-
DG2, S6K Inhibitor II	5	P70S6Ki2	1254209
Go-6976	1	PKCi1	302449
Bisindolylmaleimide-I	1	PKCi2	7463
H-1152	5	ROCKi1	406821
Y-27632	10	ROCKi2	-

Supplementary Table 2. Background network data sources. K/P-S data was assembled from multiple databases (downloaded in January 2013), mapped to *Uniprot* identifiers (UPIDs) and filtered to contain only reliable, human information. The *Human Protein Reference database* (*HPRD*)[20] gathers manually curated literature information about human protein domain architecture, PTM, interactions and disease associations. The *PhosphoSitePlus* database[12] contains information about experimentally determined PTMs on mouse and human proteins, derived either from published literature or high-throughput studies. The *Phospho.ELM* database[4] contains in vivo and in vitro experimentally determined phosphorylation sites for multiple eukaryotes, derived from the literature and multiple high-throughput datasets. The *DEPOD* database[16] contains manually curated information about active human phosphatases and their substrates, cross-referenced with colocalisation and coexpression information for additional substrate confidence. The *NetworKIN*[17] data is a predicted set of K-S interactions for human sites from *Phospho.ELM* and *PhosphoSite*, predicted using a combination of sequence motif and network context information. The *HPRD* data was mapped to UPIDs using the mapping file provided. The *PhosphoSitePlus* data was filtered to contain only human K-S interactions. The *NetworKIN* data was filtered to exclude interactions with either a motif or context score below 0.5, and predicted interactions for CSK21 and CSK22 (2288 each) were excluded because their low specificity led these kinases to dominate any network regardless of the data. The *Phospho.ELM* and *DEPOD* data were filtered for human protein substrates. Identifiers from *PhosphoSitePlus*, *NetworKIN*, *Phospho.ELM* and *DEPOD* were mapped to UPIDs using the *Uniprot* ID mapping tool, unmapped entries were manually mapped. The final bipartite K/P-S network contains 128,251 interactions between 604 K/P and 17,319 sites on 4,567 proteins. These interactions form a biologically realistic and biochemically interpretable support to model perturbation flow.

**Database**	**K/P**	**Substrate proteins**	**# interactions**
HPRD	439	1,307	313/5,869 (dephos./phos.)
PhosphoSitePlus	322	1,747	7,670 (phos.)
NetworKIN	94	3,617	91,905 (phos.)
Phospho.ELM	270	929	8,899 (phos.)
DEPOD	76	205	750 (dephos.)

Supplementary Table 3. Evaluation of the scoring scheme: categories of data points after linear model estimation of fold changes and Gaussian mixture modelling. Fold changes (FC) for drug vs control and associated adjusted p-values are estimated with the linear model, for each peptide. A GMM is fitted to each peptide and those with two components are kept. Scores Si,j are computed for each data point (peptide i, drug j) that capture the log ratio of the probability of being in the control vs perturbed distributions, using parameters estimates from the GMM. If a measurement has an S_{ij} below -0.5 (i.e. it is highly more likely to be in the perturbed state) and the FC vs control has an adjusted p-value below 0.05 (i.e. the effect of the treatment is reproducible across replicates), then we refer to it as a "double positive". If a measurement has an S_{ij} above 0.5 and an adjusted p-value for FC vs control above 0.05, then we call it a "double negative" (see Supplementary Table 3). The two metrics (FC significance and score) agree in most cases, with 78.6% of the data being correctly categorised as either double negatives or positives (DP, DN). A small proportion of peptides lie just in between the control and perturbed distribution (U, i.e. undetermined), and a small proportion of significantly perturbed peptides are misclassified as control (SN, single negative, i.e. significant FC classified in the control distribution). A larger proportion is classified as single positives (SP), which is expected with MS data as a result of relatively high FCs with low reliability (e.g. when one of the data points, either the control or the drug treated point, lie at an extreme of the dynamic range of the machine).

	**$S_{i,j}$ < -0.5**	**-0.5 < $S_{i,j}$ < 0.5**	**-0.5 < $S_{i,j}$**
**Adj.p.val < 0.05**	Perturbed,DP	U	Control, SN
**Adj. p.val. > 0.05**	Perturbed, SP	U	Control, DN
DP+DN=78.6%	SP=18.4%	U=2.3%	SN=0.7%

Supplementary Table 4. Drugs table for the independent dataset used for validation. Similarly to what is done in the main dataset, drugs are applied for an hour on MCF7 cell line cultures. Cells are then collected, proteins extracted, trypsin-digested, phospho-enriched and analysed using mass spectrometry. Drugs and their concentrations are chosen based on availability and response of canonical substrates of targeted kinases based on literature.

Drug	Concentration (μM)	Name	chEMBL ID
AKT inhibitor VIII	1	AKTi1	CHEMBL258844
AKT inhibitor X	10	(AKT inhibitor)	CHEMBL1398474
ERK inhibitor I	30	ERKi1	CHEMBL1403932
ERK inhibitor II	20	ERKi2	CHEMBL259551
GSK-1120212 (Trametinib)	0.1	MEKi1	-
U0126	10	MEKi2	CHEMBL100473
KU-0063794	2	MTORi1	CHEMBL1078983
AZD-8044	2	MTOR inhibitor	CHEMBL1801204
RSK inhibitor II	20	(P70/P90RSK inhibitor)	CHEMBL573107
PF-4708671	20	P70S6Ki1	-
SL0101	50	(P90RSK inhibitor)	CHEMBL240954
IPA-3	25	(PAK inhibitor)	CHEMBL472940
PI-103	1	PI3Ki2	CHEMBL573393
GDC-0941	1	PI3Ki1	CHEMBL521851
Go-6976	1	PKCi1	CHEMBL302449
Bisindolylmaleimide-I	1	PKCi2	CHEMBL7463
Raf inhibitor IV	0.5	(Raf inhibitor)	CHEMBL373011
ZM-336372	1	(Raf inhibitor)	CHEMBL186526

Supplementary Table 5. Predicted kinases for sites perturbed under MTOR inhibition, with no known kinase. This table contains the kinases predicted by *NetworKIN* as potentially responsible for the phosphorylation of sites found to be perturbed under MTOR inhibition, and for which no known kinase was available in other databases. By connecting these sites to the predicted kinases in our optimized network, and combining with experimental evidence of perturbation, we were allowed to prioritise 1 (or 2) kinases that are likely to be responsible for the phosphorylation of these sites, at least in the conditions observed.

Phosphorylation site	Predicted kinases	Kinase confirmed by our model
CCNL1.S.335	MK08, MK10, CDK1, CDK2, GSK3A, GSK3B, MK11, MK03, MK14, MK09, CDK5, MK12, CDK3, CDK14	CDK2
ACINU.S.1004	GSK3B, CDK2, GSK3A, CDK1, CDK5, CDK3, CDK14	CDK2
CD2AP.S.233	AKT1, KS6A1, KS6A3, KS6B1, Q0VAN0, KS6A5, G3XAA9, KS6A2, SGK1, KS6B2, AKT3, SGK3	KS6B1/AKT1
4EBP2.T.46	MK10, MK08, MK09, MK11, MK14, CDK1, CDK2, CDK5, MK03, MK12, CDK3	CDK2
MACF1.S.1376	AKT1, KS6A1, KS6B1, KS6A3, Q0VAN0, KS6A5, G3XAA9, KS6A2, KAPCA, KAPCB, SGK1, KS6B2, KAPCG, AKT3, SGK3, PRKX	KS6B1/AKT1
MYO5A.S.1652	AKT1, SGK1, KS6A1, KS6A3, KS6B1, KS6A5, Q0VAN0, G3XAA9, KS6A2, KAPCB, KAPCA, KAPCG, KS6B2, AKT3, SGK3	KS6B1/AKT1
PKCB1.S.490	CDK2, CDK1, GSK3B, CDK5, GSK3A, CDK3, CDK14	GSK3B
RPGF6.S.1070	AKT1, KS6A1, KS6A3, ATM, KS6B1, PRKDC, KS6A5, Q0VAN0, G3XAA9, KS6A2, SGK1, KS6B2, AKT3, SGK3	KS6B1/AKT1
SNUT1.S.448	KS6A1, KS6B1, KS6A3, KS6A5, G3XAA9, KS6A2, KAPCB, KAPCA, KS6B2, KAPCG, PRKX	KS6B1
SF3B1.S.129	CDK2, CDK1, GSK3B, CDK5, GSK3A, CDK3	CDK2
LAP2A.S.310	CDK2, CDK1, MK08, GSK3B, MK10, MK11, MK14, MK09, CDK5, GSK3A, MK03, MK12, CDK3	GSK3B
UBP32.S.1361	CDK1, CDK2, CDK5, CDK3, CDK14	CDK2
UNK.S.385	CDK2, CDK1, GSK3B, MK14, MK08, MK10, MK11, MK03, CDK5, GSK3A, MK09, MK12, CDK3, CDK14, CDK16	CDK2

Supplementary Table 6. Predicted kinases for sites perturbed under MTOR inhibition, with no known kinase, in the validation data set. This table contains the kinases predicted by *NetworKIN* as potentially responsible for the phosphorylation of sites found to be perturbed under MTOR inhibition in the publicly available validation data set (see Supplementary Fig. 8), and for which no known kinase was available in other databases. Note that the quality and the coverage of this data set being lower than that of the main data set, the network obtained is also less informative. However, by connecting these sites to the predicted kinases in our optimized network, and combining with experimental evidence of perturbation, we were still able, for most of these sites, to prioritise 1 (or 2) kinases that are likely to be responsible for the phosphorylation of these sites, at least in the conditions observed.

**Phosphorylation site**	**Predicted kinases**	**Kinase confirmed by our model**
RPRD2.S.665	CDK2, CDK1, CDK5, CDK3, CDK14	CDK2
ACLY.S.455	KAPCA, KAPCB, KAPCG, KS6A1, KS6A3, KS6A5, KS6B1, G3XAA9, KS6A2, KGP1, KS6B2, KAPCG, KGP2	KS6B1
EMSY.S.209	AKT1, KS6A1, KS6A3, KS6A5, KS6B1, KPCD, PKN1, Q0VAN0, KPCI, KPCT, KPCZ, G3XAA9, J3KN97, KPCG, KS6A2, KAPCB, KAPCA, KPCB, SGK1, KS6B2, KPCE, PKN2, KAPCG, AKT3, SGK3, KPCL	KS6B1, AKT1
FLNA.S.1459	GSK3B, CDK5, MK08, CDK2, GSK3A, MK10, CDK1, MK11, MK14, MK09, MK03, MK12, CDK3	?
KIF4A.S.801	KS6A1, AKT1, KS6A3, KS6A5, KS6B1, Q0VAN0, G3XAA9, KS6A2, KAPCB, KAPCA, KGP1, SGK1, KS6B2, KAPCG, AKT3, KGP2, SGK3, PRKX	KS6B1, AKT1
TP53B.S.1317	KS6A1, KS6A3, KS6A5, KS6B1, KPCD, PKN1, KPCI, KPCT, KPCZ, G3XAA9, J3KN97, KPCG, KS6A2, KAPCB, KAPCA, KPCB, KS6B2, KPCE, PKN2, KAPCG	?
NIPA.S.407	AKT1, KS6A1, KS6A3, KS6A5, KS6B1, Q0VAN0, G3XAA9, KS6A2, KAPCB, KAPCA, KGP1, SGK1, KS6B2, KAPCG, AKT3, KGP2, SGK3	KS6B1, AKT1

Supplementary Table 7. Prediction of drug targets using *PHONEMeS*. This table details the analyses that were done using *PHONEMeS* and individual drugs as well as pairs of drugs with the same nominal target(s), in order to investigate kinase inhibitors specificity, efficacy and effect on the phosphoproteome. The full resulting networks are available on http://www.cellnopt.org/phonemes. This table shows the number of nodes and edges in the background network for each analysis, the number of sites perturbed under each drug, on which these background networks are based, and the scores (final generation population averages and best models) for each of three replicate optimisations, when multiple set ups are to be compared.

Name	Drugs	Targets (drug 1/drug2)	Size (edges/nodes)	# sites P	Scores (pop. avg)<br>Drug1<br>Drug2	Scores (best model)<br>Drug1<br>Drug2
AKTi1	AKTi1	AKT1, AKT2	2731/734	123		
AKTi2	AKTi2	AKT1, AKT2, AKT3	4352/ 1021	71		
AKTia	AKTi1/AKTi2	AKT1, AKT2 / AKT1, AKT2, AKT3	4915 / 1093	123/71 (61 common)	9.9, 11.5, 12.5<br>5.5, 5.6, 6.1	-3.6, -2.0, -2.3<br>3.5, -4.0, -2.8
AKTib	AKTi1/AKTi2	AKT1, AKT2	2792/741	123/71 (61 common)	6.5, 6.2, 9.0<br>3.0, 3.1, 4.7	8.0, -7.2, -5.2<br>6.1, -5.8, -5.7
MEKi1	MEKi1	MP2K1, MP2K2	1432/463	10		
MEKi2	MEKi2	MP2K1, MP2K2	1313/426	8		
MEKi	MEKi1/MEKi2	MP2K1, MP2K2	1506/484	10/8 (4 common)		
CAMKi1	CAMKi1	KCC2A, KCC2B, KCC2G	2349/661	35		
CAMKi2	CAMKi2	KCC2A, KCC2B, KCC2G	2012/612	24		
CAMiK	CAMKi1/CAMKi2	KCC2A, KCC2B, KCC2G	2720/751	35/24 (13 common)		
EGFRi1a	EGFRi1	EGFR	684/294	22	0.82, 0.96, 0.81	-1.43, -1.57, -1.54
EGFRi1b	EGFRi1	EGFR, ERBB2	778/332	22	0.70, 0.58, 0.62	-1.50, -1.38, -1.42
EGFRi2	EGFRi2	EGFR	536/251	13		
EGFRia	EGFRi1/EGFRi2	EGFR	742/304	22/13 (6 common)	0.70, 0.69, 0.66<br>0.50, 0.57, 0.60	-1.59, -1.51, -1.68<br>-1.68, -1.73, -1.76
EGFRib	EGFRi1/EGFRi2	EGFR, ERBB2 / EGFR	836/342	22/13 (6 common)	0.61, 0.55, 0.60<br>0.85, 0.64, 0.74	-1.52, -1.59, -1.27<br>-1.59, -1.75, -1.69
ERKi1a	ERKi1	MK01	539/230	20	0.58, 0.78, 0.65	-0.42, -0.42, -0.46
ERKi1b	ERKi1	MK01, MK03	2020/564	20	0.49, 0.53, 0.52	-0.36, -0.36, -0.41
ERKi1c	ERKi1	MK01, MK03, MK14	2255/627	20	0.42, 0.45, 0.32	-0.42, -0.46, -0.40
ERKi2a	ERKi2	MK01	721/330	21	0.72, 0.79, 0.82	-0.87, -0.84, -0.85
ERKi2b	ERKi2	MK01, MK03	2534/703	21	0.64, 0.51, 0.93	-0.51, -0.65, -0.76
ERKi2c	ERKi2	MK01, MK03, MK14	2891/776	21	0.93, 0.40, 0.67	-0.52, -0.80, -0.57
ERKi2d	ERKi2	MK03	972/383	21	0.24, 0.27, 0.22	-0.91, -0.83, -0.94
ERKia	ERKi1/ERKi2	MK01	886/346	20/21 (4 common)	1.09, 0.96, 0.70<br>0.95, 0.87, 0.60	-0.15, 0.038, 0.0078<br>-0.52, -0.77, -

						0.77
ERKib	ERKi1/ERKi2	MK01 / MK01, MK03	2700/719	20/21 (4 common)	0.75, 0.77, 0.98<br>0.83, 0.86, 0.43	0.37, 0.25, -0.0047<br>-0.45, -0.56, -0.66
ERKic	ERKi1/ERKi2	MK01/MK01, MK03, MK14	3062/792	20/21 (4 common)	0.91, 0.84, 0.85<br>0.78, 0.86, 0.97	0.44, 0.28, 0.18<br>-0.42, -0.39, -0.55
ERKid	ERKi1/ERKi2	MK01, MK03, MK14 / MK03	3062/792	20/21 (4 common)	0.54, 0.44, 0.53<br>0.72, 0.43, 0.64	-0.069, 0.046, -0.17<br>-0.35, -0.46, -0.50
MTORi1	MTORi1	MTOR	840/309	30		
MTORi2	MTORi2	MTOR	1002/361	30		
MTORi	MTORi1/MTORi2	MTOR	1028/365	30/30 (26 common)		
PI3Ki1a	PI3Ki1	PK3CA, PK3CD, MTOR	3713/948	34	1.94, 1.58, 1.64	-2.04, -1.92, -2.31
PI3Ki1b	PI3Ki1	PK3CA, PK3CD, MTOR, AKT1	3713/948	34	0.85, 0.86, 0.92	-2.45, -2.18, -2.27
PI3Ki2a	PI3Ki2	PK3CA, PK3CD	3556/960	40	4.35, 4.34, 4.65	-2.68, -2.85, -2.71
PI3Ki2b	PI3Ki2	PK3CA, PK3CD, AKT1	3570/952	40	1.93, 1.01, 1.30	-2.18, -3.38, -3.05
PI3Ki2c	PI3Ki2	PK3CA, PK3CD, MTOR	3566/952	40	2.19, 1.77, 2.34	-2.50, -2.25, -2.57
PI3Kia	PI3Ki1/PI3Ki2	PK3CA-D, MTOR / PK3CA-D	3952 / 1001	34/40 (29 common)	1.83, 1.75, 1.89<br>4.18, 4.10, 4.19	1.81, -1.85, -2.11<br>-1.81, -1.91, -2.26
PI3Kib	PI3Ki1/PI3Ki2	PK3CA-D, MTOR, AKT1 / PK3CA-D, AKT1	3952 / 1001	34/40 (29 common)	0.89, 1.38, 0.78<br>1.28, 1.68, 1.17	-2.27, -2.11, -2.43<br>-2.39, -1.90, -2.32
P70S6Ki1a	P70S6Ki1	KS6B1	853/364	26	1.57, 1.22, 1.23	-2.00, -1.86, -2.13
P70S6Ki1b	P70S6Ki1	KS6B1, KS6B2	2292/709	26	0.95, 1.10, 2.33	-1.85, -1.73, -1.38
P70S6Ki2	P70S6Ki2	KS6B1	951/362	34		
P70S6Kia	P70S6Ki1/P70S6Ki2	KS6B1	1133/425	26/34 (16 common)	0.94, 2.26, 1.66<br>0.66, 1.44, 1.134	-1.99 -1.25, -1.66<br>-0.83, -0.21, -0.41
P70S6Kib		KS6B1, KS6B2 / KS6B1	2809/787	26/34 (16 common)	1.08, 0.96, 1.43<br>1.28, 0.93, 1.35	-1.48, -1.63, -1.61<br>-0.67, -0.69, -0.67
PKCi1a	PKCi1	KPCA, KPCB	2216/689	26	0.57, 0.58, 0.45	-0.51, -0.51, -0.57
PKCi1b	PKCi1	KPCA, KPCB, KPCG, KPCE	2791/805	26	0.50, 0.60 0.47	-0.38, -0.61, -0.48
PKCi2a	PKCi2	KPCA, KPCB, KPCG, KPCE	2088/662	12	0.18, 0.29, 0.17	-0.41, -0.42, -0.41
PKCi2b	PKCi2	KPCA-B-G-E-T,GSK3B	2270/687	12	0.33, 0.21, 0.14	-0.37,4 -0.43, -0.44
PKCia	PKCi1/PKCi2	KPCA-B / KPCA-B-G-E	3119/869	26/12 (2 common)	0.70, 0.54, 0.45<br>0.48, 0.35, 0.25	-0.50 -0.27, -0.62<br>0.11, -0.084, -0.037
PKCib	PKCi1/PKCi2	KPCA-B-G-E / KPCA-B-G-E-T,GSK3B	3264/877	26/12 (2 common)	0.42, 0.42, 0.43<br>0.46, 0.38, 0.41	-0.30, -0.39, -0.45<br>0.010, -0.029, -0.12
ROCKi1a	ROCKi1	ROCK1	1063/419	24	1.09, 0.94, 0.96	-0.62, -0.57, -0.62

ROCKi1 b	ROCKi1	ROCK1, KAPCA, KAPCB, KAPCG	1868/572	24	0.32, 0.27, 0.59	-0.90, -0.90, -0.68
ROCKi2 a	ROCKi2	ROCK1	874/374	10	0.37, 0.37, 0.39	-0.51, -0.50, -0.50
ROCKi2 b	ROCKi2	ROCK1, KPCE	1850/575	10	0.24, 0.095, -0.034	-0.50, -0.49, -0.51
ROCKia	ROCKi1/ROCKi2	ROCK1	1094/421	24/10 (7 common)	0.96, 0.97, 1.02<br>0.41, 0.43, 0.46	-0.57, -0.60, -0.16<br>-0.095, -0.079, -0.34
ROCKib	ROCKi1/ROCKi2	ROCK1, KAPCB-A-G / ROCK1,KPCE	2621/719	24/10 (7 common)	0.47, 0.51, 0.68<br>0.16, 0.23, 0.28	-0.61, -0.61, -0.64<br>0.38, -0.47, -0.27

Supplementary Notes

Supplementary Note 1

Proof of principle on data with reduced scope

In order to test our method, we reasoned that it should be able to recover a canonical kinase hierarchy when exposed to a reduced dataset that is consistent with this hierarchy. Therefore, we built and trained models based on the data for 6 small-molecule inhibitors (in pairs targeting class I PI3-kinases, Akt and mTOR) and 19 sites whose behaviours are consistent with the canonical view that PI3K leads to the activation of Akt which is then responsible for the activation of mTOR (Supplementary Fig. 3a-b, f), such that sites affected by inhibition of mTOR or Akt should also be affected by inhibition of PI3K etc. Twenty-three additional sites that lie on potential paths between the 19 perturbed sites and the drug targets were included in the problem at the automatic background network-building step (Supplementary Fig. 3c). We also used this setting to study the influence of all free training parameters in order to determine the reasonable ranges to use (Supplementary Fig. 4). This approach does not impose any constraint on noise, function or identity of the sites selected. It does however allow for the presence of contradictory and uncertain information on paths between targets and data sites, which would unavoidably occur in a real setting.

Three independent trainings were combined into a single solution (Supplementary Fig. 3d). The resulting network contained 39 nodes (all referred to by their *Uniprot* identifiers) and 36 edges with 0 % tolerance (12 and 6 % of the total nodes and edges in the background network, respectively), and 45 nodes and 49 edges (15 and 8 %) with 20 % tolerance (Supplementary Fig. 3e). This network robustly captures the expected hierarchy of inhibited kinases (even with noisy and inconsistent data) and the perturbation of many of their canonical substrates (Fig. 3e). Indeed, the solutions correctly recovers the PK3CA->AKT1->MTOR cascade and connects the majority or sites to these or downstream kinases, including sites that are not direct targets of any of the inhibited kinases. It also captures perturbations of "unexpected" sign (i.e. phosphorylation increased upon kinase inhibition), such as the increase in DPYL2-pT509, which is explained in the model by the decrease in phosphorylation of the inhibitory GSK3B-pS9. The model cannot fully capture some sites found perturbed under PI3K inhibition (SRPK1-pS51, HNRPC-pS253 and possibly CDK16-pS153 and SRRM1-pS769), which is an expected consequence of PI3K being primarily a lipid kinase and not a protein kinase (whereas our background network only covers protein substrates). Within these limitations, *PHONEMeS* has demonstrated its ability to recover a canonical kinase signaling hierarchy when the data comply with its implied signal flow. Therefore, using unfiltered phospho-MS data with *PHONEMeS* should allow us to study non-canonical aspects of kinase inhibitions and resulting downstream perturbations.

Supplementary Note 2

Single drugs analysis: efficacy and specificity in context

The data set analysed here contains drug treatments in pairs with the same nominal target(s), to increase confidence in the data by focusing on perturbations observed under both drugs in a pair (hence reducing the effects of noise and off target effects). However, there are two reasons for a drug pair to show a different set of perturbed sites: i) the two drugs have slightly different targets (or off target) effects, ii) a slightly different set of sites under the same targets are detected and/or reach significance. Secondary targets are common and often poorly characterised, especially when studying kinase inhibition in cells rather than in solution. However, when looking at shotgun MS data, option ii) is also a plausible explanation for differences between drugs. Using *PHONEMeS*, we can look at the efficacy and specificity of drugs in a pair in the presence of both of these sources of uncertainty. In order to do this, we separately optimised a network for each drug treatment and each pair of drug treatments, using different sets of potential targets based on the drugs' nominal targets and secondary targets mentioned by the manufacturer, and secondary targets found in *chEMBL* (see Supplementary Table 7).

Because additional targets can make the background network significantly more complex (see table Supplementary Table 7), leading to less stable and less determined solutions, we decided to be relatively conservative in adding secondary targets. In addition, in some cases adding a target to one drug in a pair will allow to explain sites that are different between two drugs in a pair, regardless of whether they result from actual differences in targets or just in detection of the perturbations. However, if there really is a difference in drug specificity, then adding the target to the individual drug optimisation should also lead to significant score improvements. Additionally, when the candidate target is already pulled in a network where it is not specified as a target (i.e. it seems necessary to explain the data) we can be more confident that the candidate kinase is a "real" target of the drug. Finally, when interpreting the results of this analysis, it is important to remember that drugs can show widely variable effects on the network both in terms of spread (how far they reach) and breadth (how many areas of the network they hit) for multiple reasons: i) if the kinase inhibited is highly specific to a limited number of substrates, the breath of the perturbation will accordingly be small; ii) we are looking at a single snapshot in time, hence when the signalling spreads slowly in the network, it is possible that the full reach of an inhibition has not yet been realised; iii) drugs can have partial inhibitory activity, which is difficult to capture in a Boolean setting; iv) we are looking exclusively at inhibition compared to the basal state, hence we will not see anything in the network if the kinase was not significantly activated in the basal state.

All optimisations were performed as described in the main text. The results of all of these analyses can be found in table Supplementary Table 7 and the associated resulting networks can be downloaded as *Cytoscape* workspaces on the *PHONEMeS* webpage http://www.cellnopt.org/phonemes. Some of these analyses are discussed in detail below.

Akt Akt (or protein kinase B) is a serine/threonine kinase that regulates key cellular processes from cell growth to survival. Mammalian cells express three different isoforms of Akt (Akt1,2,3) with close specificity but different tissue-specific patterns of expression[7, 11]. Akt1 activation by growth factors is relatively well known and relies on the membrane recruitment of Akt via its pleckstrin homology (PH) domain which interacts with phosphoinositides PI(3,4)P2 and PI(3,4,5)P3 produced by activated phosphatidylinositol 3-kinase (PI3K). This interaction enables phosphorylation of Akt in its activation loop at T308 by PDPK1. mTORC2 or DNA-PK phosphorylation of Akt at S473 results in a fully activated Akt[7, 11]. Activated Akt translocates to the cytosol and nucleus, where it phosphorylates many substrates.

AKTi1 is a PH domain-dependent inhibitor of Akt1,2 (and Akt3 to a lesser extent), and AKTi2 is an allosteric non-ATP competitive inhibitor of Akt1,2 and 3. Both inhibitors are, according to manufacturer information, specific inhibitors of these targets, which their *chEMBL* records seem to confirm. However, AKTi1 treatment results in many more sites perturbed than the AKTi2 treatment. As we can see from table Supplementary Table 7 and Supplementary Fig. 10, the score (for both drugs) are much better when Akt1 and Akt2 are used as common targets of both drugs (final population average scores improved from 11.3±1.3 to 7.2±1.5 for AKTi1 and 5.7±0.3 to 3.6±0.9 for AKTi2), and the models are also much smaller when Akt3 is not included (final population average model size from 892/892/894 to 591/592/615 edges). Additionally, even when Akt3 is included in the AKTi2 optimisation, few sites (2 out of 71) are actually placed under Akt3 (not shown), indicating that Akt3 is probably not necessary to explain the data. This would suggest that either Akt3 is not significantly inhibited by the drugs, or it is simply not significantly active (or even expressed) in this cell line under these conditions. Finally, in the combined optimisations, we did not see sites perturbed under only one of the two drugs clustering under any specific kinase, indicating that the difference between the two drugs are more likely to result from a difference in detection rather than specificity (see Supplementary Fig. 10). The fact that, for a lot of sites perturbed only under AKTi1, the sign of the fold change under AKTi2 is consistent with the one under AKTi1 but just doesn't reach significance, supports this hypothesis (see Supplementary Fig. 10). Together, these results suggest that both AKT inhibitors are best represented by inhibition of Akt1 and Akt2.

MEK MAPK/ERK kinase (MEK) 1 and 2 are two closely related dual specificity protein kinases, part of the mitogen-activated protein kinase (MAPK) cascades which are involved in regulation of cell proliferation, survival and differentiation. MEK 1/2 are activated by the Raf serine/threonine kinases (c-Raf-1, A-Raf, B-Raf), which are themselves activated primarily by Ras small GTPases (although additional signalling activities through PAK, Src, PP2A, 14-3-3 proteins etc have been shown to play a role in Raf activation). The Ras proteins (H-, K- and N-Ras) are activated by extracellular stimuli such as EGF through the epidermal growth factor receptor (EGFR). MEK1 and 2 phosphorylate serine/threonine and tyrosine residues of their only known substrates ERK1/2 (extracellular signal-regulated kinase 1/2). Activated ERKs regulate a large number of (mainly nuclear) proteins. Mutations and/or overexpression at multiple levels of this cascade are frequently implicated in a variety of cancers[21].

MEKi1 is a selective allosteric inhibitor of Mek1,2 kinase activity, and MEKi2 is a specific non-competitive (with respect to ATP and Erk) inhibitor of Mek1,2. As we can see from Supplementary Table 7, these drug treatments result in the lowest number of perturbed sites of all drugs tested. Given the narrow substrate specificity of the MEK kinases and the high specificity of their inhibitors[21], it is not overly surprising that the number of sites perturbed is relatively small, although we would have perhaps expected to see more of the downstream effects via substrates of ERK. It is possible that the time frame used in this experiment did not allow for this to appear quite strongly enough to be consistently detected. Nonetheless, the reconstructed paths found here accurately reflect the MEK-ERK-JNK expected signalling flow and show defined paths to plausible effectors of the pro-survival activity of this pathway. Both drugs have the expected result of a decrease in phosphorylation of MK01-Y187 and MK03-Y204, which are activating sites of Erk1 and Erk2, respectively (see Supplementary Fig. 11.). Paths of interest include the decrease in phosphorylation of B2L11-S77, which is an inducer of apoptosis, under MK10-T221 (which is an activating site of MK10/JNK3, and the decrease of B2L11-S77 is therefore consistent with this path). This site is not recorded as functional in *Uniprot* but interestingly S69 is reported to be phosphorylated by Erk1,2, leading to proteasomal degradation of B2L11. The decrease in phosphorylation of TR10A.S466 (reached via MK03 and KC1E) is also of interest since this protein is the receptor for the cytotoxic ligand TRAIL, and therefore also plays a role in induction of apoptosis.

ERK Erk1/2 are terminal serine/threonine kinases of the MAPK cascades activated by growth factor-stimulated cell surface receptors, such as receptor tyrosine kinases (RTKs) of

EGF and insulin[2, 21]. Upon activation by MEK1/2 (see above), ERKs can translocate to the nucleus where they regulate a variety of transcription factors, leading to changes in cell proliferation, survival and motility. Erk1/2 signalling can also regulate cytoplasmic substrates like TSC2, RSK, members of the BCL2 family of apoptotic regulators, etc. Erk1/2 also exert negative feedback effects at multiple levels in the MAPK cascade[2, 18].

ERKi1 is a non-ATP-competitive erk1/2 inhibitor that prevents interaction with substrates by binding erk docking domains. It preferentially binds erk2, and has little effect on erk1/2 phosphorylation by mek1/2[10]. ERKi2 is a potent ATP-competitive inhibitor of erk1/2 (MK01/MK03), which according to *chEMBL* also has activity towards MK14. As we can see in Supplementary Table 7, the two drugs only have four perturbed sites in common. Considering that the two drugs have different modes of action and partially overlapping known targets, this is perhaps not overly surprising but constitutes a sure warning sign. While performing the separate optimisations for ERKi1 and ERKi2, we noticed that the ERKi1 optimisation performed best when MK14 is added as an off-target of the drug (final population average score from 0.52±0.02 to 0.40±0.07, see Supplementary Table 7. Additionally, MK14 was pulled into the solution networks when it was not specified as a target, with direct substrates showing a decreased phosphorylation level (see Supplementary Fig. 12). On the other hand, the ERKi2 optimisations showed no improvement upon addition of MK14 as a target (final population average score from 0.69±0.22 to 0.67±0.27, and MK14 was not pulled into the network when it was not specified as a target. Instead, it is the optimisation with MK03 only that performs best (final population average score of 0.24±0.02, see Supplementary Fig. 12 and Supplementary Table 7. It would therefore seem that the identity of the two drugs was swapped somewhere in the process of generating or recording the data. As we can see on Supplementary Fig. 13, optimising with MK01, MK03 and MK14 as targets of ERKi1, and MK03 as a target of ERKi2 performed best in terms of scores (final population average score of 1.1±0.2 compared to 1.5±0.1, 1.7±0.4 and 1.7±0.1 for the other setups, see Supplementary Table 7 but also allows placing of perturbed sites in a much more consistent way (compare the networks on the left and on the right on Supplementary Fig. 13: sites found to be perturbed only under ERKi2 are mostly placed under MK03, whereas many sites found to be perturbed exclusively under ERKi1 are placed under MK14). This setup manages to relatively successfully explain some of the large differences in effect of the two drugs that we have seen above.

PI3K The PI3Ks are lipid kinases activated by growth factor receptor tyrosine kinases (RTK) and G-protein-coupled receptors (GPCRs). PI3Ks phosphorylate phosphatidylinositol-4,5-bisphosphate (PIP2) to produce phosphatidylinositol-3,4,5-trisphosphate (PIP3). Phosphatidylinositol is a membrane phospholipid that can be phosphorylated at the 3, 4 and 5 positions of the inositol ring, generating species with different interaction properties and signalling outcomes (see *Bunney et al.*[1] for a review). PIP3 recruits to the plasma membrane and activates several PH domain-containing proteins such as PDK1 and AKT, driving cell cycle progression and survival[5, 8, 19]. Class IA PI3Ks are heterodimeric lipid kinases containing a p110 catalytic subunit (PIK3CA, PIK3CB and PIK3CD) and a p85 regulatory subunit. PIK3CA mutations are the most common genetic alterations of this pathway in breast cancer (Miller et al., 2011), and indeed the cell line used here, MCF7, harbours a E542K mutation within the helical domain of p110α (see http://cancer.sanger.ac.uk/cosmic/mutation/overview?id=760). Such mutations seem to abrogate the inhibitory interaction between p85 and p110, hence the mutant p110α subunits are known to increase *in vitro* lipid kinase activity and, in some cases, to maintain PI3K-Akt signalling under growth-factor deprivation[5]. Cancer cells expressing those mutants are however similarly susceptible to PI3K inhibitors as those expressing wild type PI3K[3], and studies in patients seem to indicate that such mutations confer increased sensitivity to PI3K/Akt/mTOR inhibitions, possibly indicating a dependency of these cancers on this pathway[14].

PI3Ki1 is a potent ATP-competitive pan-PI3K inhibitor (equally potent against oncogenic mutants of p110α)[19]. On top of the nominal targets (PK3CA, PK3CB, PK3CG, PK3CD, *chEMBL* records AKT1 and MTOR as targets of this inhibitor. PI3Ki2 is an ATP-competitive inhibitor of PI3K, mTOR and DNA-PK. Small molecule inhibitors of p110 often also inhibit mTOR due to structural similarities. The *chEMBL* record for PI3Ki2 only lists interaction with AKT1.

When optimising with PI3Ki1 alone, the setup with AKT1 and MTOR as off targets produced the best scores (final population average score of 0.88±0.04 vs 1.72±0.19, see Supplementary Table 7) and the decrease of phosphorylation at many direct targets of these two kinases would confirm that they are affected by the drug (see Supplementary Fig. 14, left). Since these two kinases are the main downstream targets of activated PI3K, it is also possible that the improvement in the optimisation comes from the fact that declaring those as targets simplifies the search by automatically connecting their perturbed substrates. A similar situation is observed for PI3Ki2, where adding AKTi1 as an off target drastically improved the scores from final population average scores of 4.45±0.17 to 1.42±0.47, see table ST.7. MTOR was pulled into the network whether it was declared as a target or not, and adding it as a target also improved the scores to 2.10±0.29 (see Supplementary Fig. 14, right and Supplementary Table 7). In comparison, specifying AKT1 as a target did improve the scores (see above), but it seemed to do so mainly by explaining its direct substrates. This would indicate that both drugs have PI3K (PK3CD and PK3CA) and MTOR as main targets. The combined network scores were drastically improved by adding off targets MTOR and AKT1, especially for PI3Ki2 (final population average scores from 1.82±0.07 to 1.01±0.32 for PI3Ki1 and 4.16±0.05 to 1.38±0.27 for PI3Ki2, see Supplementary Table 7. The precise connectivity between MTOR, AKT, PI3K and KS6B1 is somewhat unclear because most configurations are equivalent as far as the model is concerned, but there is clearly an effect of both drugs on MTOR and AKT1 (either directly, which seems likely for MTOR, or via PI3K/MTOR inhibition, which seems more likely for AKT). Beyond the clear effect on MTOR and AKT, the networks suggest an important role for the kinase SRC.

P70S6K The 70kDa ribosomal protein S6 kinases (S6K1 and S6K2, collectively P70S6K) are serine/threonine kinases of the AGC family (which also includes Akt, PKCs, P90RSK, etc). The catalytic domains of S6K1 and S6K2 are very similar but differences in the extreme C- and N-terminal regions are thought to lead to different localisation and target properties. The S6Ks are primarily activated through the PI3K pathway. The physiological target of S6Ks is the ribosomal S6 protein (rs6p, a component of the 40s ribosomal subunit), the phosphorylation of which plays a key role in modulating translational efficiency. S6Ks also directly phosphorylate initiation (e.g. eiF4B) and elongation (e.g. eEF2k) factors, as well as other proteins involved in proliferation and survival. Additionally, S6Ks seem to play an important role in mediating negative feedback loops in the PI3K signalling network[6].

P70S6Ki1 is an ATP-competitive S6K1-specific inhibitor that was shown to prevent S6K1-mediated phosphorylation of rs6p. Both the single drugs and combined drugs network (see Supplementary Fig. 15 and Supplementary Fig. 16) look cleaner when only KS6B1 (S6K1) is used as a unique common target of the two drugs, although the score curves are remarkably variable (final population average scores of 1.46±0.76 and 1.34±0.20 for the P70S6Ki1 optimisations, 2.35±0.45 and 2.70±1.05 for the combined optimisations, respectively with and without KS6B2, see Supplementary Table 7). This makes it hard to conclude anything on the scores differences, especially since the problem is worsened by a doubling of the size of the networks after adding S6K2 as a target (background network from 853 to 2292 edges for P70S6Ki1 optimisations, and 1133 to 2809 edges for the combined optimisations, see Supplementary Table 7). Despite differences in sites perturbed, many paths found downstream of P70S6Ki1 are also found under P70S6Ki2 (compare Supplementary Fig. 15 top *vs.* bottom), and there does not seem to be a clear clustering of sites specific to one or the other drug under specific kinases in the combined network (Supplementary Fig. 15), further indicating that the two drugs are likely to have the same target. Unfortunately, beyond the

direct effect on RICTR and the apparent feedback on PDK1 (via an increase in its autophosphorylated activation site S241) on which the two drugs agree, the paths observed are fairly indirect and unstable. This seems to be a consequence of the fact that many of these sites are connected to predicted rather than known kinases (which are less specific links and hence typically less stable in the optimisation). It is therefore possible that, in addition to a far from perfect agreement between the data related to the two drugs, these networks also suffer from a relative lack of precise knowledge about substrates of S6K1 (see *Fenton et al.*[6] for a review).

Supplementary Note 3

Analysis of a published unrelated MS data set

In order to prove the general applicability of our method, we went on to analyse a published data set[13]. This data set consists of iTRAQ labelled phosphoproteomic samples after 1 hour treatment of HEK-293E cells with 100 nM rapamycin, 250 nM Torin1, or DMSO followed by 20 minutes stimulation with 150 nM insulin. The data (supplementary table S2 of *Hsu et al.*[13]) consists of drug treatment vs insulin log2 ratios in biological duplicates, across 3883 peptides of which only the 1609 having data for both replicates were kept. This data was centred and standardised by replicate, and the replicate measurements were averaged for each peptide. The *Uniprot* identifiers of these peptides were obtained by mapping the *RefSeq* identifiers provided using the *Uniprot* ID mapping tool (http://www.uniprot.org/uploadlists/).

This data is much less suited for quantitative modelling of phosphorylation networks than the data that we generated ourselves. The limited number of replicates available means that it is not possible for us to obtain statistically meaningful estimates of the effect of the treatments on each peptide. Additionally, because only two treatments are applied and the data is by nature in the form of ratios rather than absolute values for each treatment and a matched control, we cannot estimate a perturbed and a control state for each phosphorylation site with a Gaussian mixture model, as we would ideally do. However, the scores used for the optimisation step are derived from the Gaussian mixture model estimates (i.e. they capture how much more likely a peptide is to be in the perturbed rather than the control state under a certain condition). We therefore had to adapt the scoring procedure in order to assign to each peptide under each of the two treatments a score that is negative if the peptide is more likely to be perturbed, and which decreases as the amplitude of the difference from the control increases. In their analysis, Hsu et al. use peptides that are 2.5 median absolute deviation below the average of the condition to define the set of peptides that are affected by a drug (under the assumption that most peptides are not expected to react to a treatment when using high-content untargeted data). As a consequence, they are essentially selecting the peptides that show a large decrease in phosphorylation under torin or rapamycin treatment, compared to the insulin (DMSO) control.

We are however interested in peptides that show an increase in phosphorylation upon treatment because our method can (and aims to) capture more than direct targets of mTOR. Nonetheless, we wanted to stay close to the strategy used by *Hsu et al.*[13], in order to be able to put our results in perspective with theirs. Therefore, we decided to use the following scoring scheme: the score of a peptide under a drug treatment (rapamycin or mTOR compared to insulin control) is $-\log_{10}(p/1-p)$ where p is the probability of obtaining a value further from the mean of the distribution for that drug treatment. As required for our optimisation scheme, this value is negative and its absolute value increases as the effect observed moves further from the mean. However, whereas our score can be negative (if a data point is more likely to belong to the perturbed distribution) or positive (if a data point is more likely to belong to the control distribution), this score is always negative because the above ration is always smaller or equal to 1. This problem can be handled by the other element that is used in our optimisation scheme, which is the assignment of each data point to the control or perturbed cluster. In order to stay close to the *Hsu et al.*[13] analysis, we assigned the “P” or “C” status based on a 2.5 MAD distance from the average of the condition. The status of this assignment is further called single negative/single positive if the two replicate measurements were too far from each other, in order to exclude clearly unreliable peptides, in the absence of a proper measure of significance. This is done by filtering the peptides using a threshold on the sum of the differences between the replicates in a pair divided by their mean. A threshold of 10 (arbitrarily chosen to exclude the most unreliable peptides while preserving a reasonable amount of data) led to a total of 1042 peptides (i.e. 567 peptides were classified as single negatives/positives).

As mentioned above, mTOR functions as part of two different complexes, mTORC1 and mTORC2. mTORC2 signalling is insensitive to nutrients but responds to growth factors such as insulin via PI3K. mTORC2 directly activates Akt by S473 phosphorylation, as well as serum-and glucocorticoid-induced protein kinase 1 (SGK1, which controls ion transport and growth), and protein kinase C α (PKCα, which is involved in regulation of cell shape) (Laplante and Sabatini, 2012). mTORC1 integrates inputs from growth factors, stress, energy status, oxygen and amino acids. The heterodimer tuberous sclerosis 1 and 2 (TSC1 and 2) is a key upstream regulator of mTORC1, which can be directly phosphorylated and inactivated by Akt and ERK1/2, resulting in mTORC1 activation. Akt also activates mTORC1 by phosphorylating PRAS40 (AKTS1), an mTORC1 inhibitor, hence causing its dissociation from raptor (an mTORC1 subunit). TNFα, Wnt signalling, DNA damage and amino-acids are also known to regulate mTORC1 activity. mTORC1 promotes protein synthesis through phosphorylation of eukaryotic translation initiation factor 4E-binding protein 1 (4E-BP1) and S6 kinase 1 (S6K1), amongst others. mTORC1 also controls the synthesis of lipids necessary for membrane biogenesis and negatively regulates autophagy and lysosomes biogenesis[15].

One of the most interesting aspects of the *Hsu et al.*[13] data is that it uses two mTOR inhibitors that are known to have a different functional effect on the kinase. Indeed, the authors used Torin1, which is an ATP-competitive mTOR kinase domain inhibitor, in parallel with rapamycin, which is an allosteric mTORC1 inhibitor. This is particularly relevant since feedback activation of the PI3K-Akt pathway upon mTORC1 inhibition is known to occur, and to be a potential problem for uses of mTOR inhibitor as anti-proliferation agents e.g. in cancer. However relevant, these problems are poorly characterised due to the complex relationships between the activities of the two complexes and their inhibitors. We therefore decided to model the effect of the two inhibitors (rapamycin and Torin1) independently, to see if we could learn something in particular about the effects of mTOR inhibition as part of mTORC1. The resulting networks (6 combined parallel optimisations, parameters as above, displayed with 20% tolerance around the best incoming edges) are displayed on Supplementary Fig. 17.

As we can see on Supplementary Fig. 17, a number of elements are common with our own analyses of mTOR inhibition, such as expected effects on KS6B1, RPTOR, AKT1, and the effect on CDK2 discussed above. More interestingly, a number of elements can only be found under Torin1 inhibition of mTOR, and are therefore presumably consequences of the inhibition of mTORC2 activity. These are: the decreased phosphorylation of AKTS1-T246 via AKT1 (as mentioned above, mTORC2 normally activates Akt which then phosphorylates AKTS1 leading to activation of mTORC1), the decreased phosphorylation of the SGK1 substrate NDRG1-S330 (SGK1 being a canonical downstream effector of mTORC2 activation), and the decreased phosphorylation of AKT3-S472. Of course, a number of elements are also common between the rapamycin and Torin1 treatments, which is expected since both inhibit mTORC1 (and feedbacks between mTORC1 and 2 are complex). Most of these common elements can be directly related to the inhibition of mTORC1. They are: the expected decreased phosphorylation of KS6B1-S447 and RPTOR-S863, the relationship between mTORC1 and DNA damage (as evidenced by the decreased phosphorylation of NPM-S70 and P53—S315), and the decreased phosphorylation of the translation inhibitor PDCD4. More interestingly, there are also a series of elements that are only observed under rapamycin inhibition of mTORC1. These are mostly increases in phosphorylation and were missed by Hsu et al. because increase in phosphorylation are difficult to apprehend when looking at direct effects of kinase inhibition (inhibition is expected to produce decreased phosphorylations), rather than putting phosphorylation perturbations in their network context. These are: the increased phosphorylation of AKT3-S472, of the cell cycle regulator APC1-S688, of the replication associated DNA ligase DNLI1-T233, and of the proteins ELAV1 and F122A. The results in Supplementary Fig. 17B provide evidence for the activation of the MAP kinases cascade and proliferative pathways upon mTORC1, which is not observed under combined mTORC1 and mTORC2 inhibition.

This analysis shows how *PHONEMeS* can be used to analyse the effects of inhibitors with partially overlapping target spectrum. The analysis both recapitulates expected relationships between the inhibitors and their expected targets (clearly placing the known downstream effects of these), and proposes hypotheses for the known phenotypic effects of these inhibitions that have very important consequences for a potential pharmacological use. More generally, this analysis demonstrates the flexibility of our method is flexible and shows that it can in principle be applied to and provide useful insights from any type of untargeted mass spectrometry phosphoproteomics data collected before and after perturbation.

Supplementary References

1. Bunney T D and Katan M. Phosphoinositide signalling in cancer: beyond PI3K and PTEN. *Nature Reviews Cancer* 10(5):342–352 (2010).

2. Cargnello M and Roux P R. Activation and function of the MAPKs and their substrates, the MAPK-Activated protein kinases. *Microbiology and Molecular Biology Reviews* **75**(1):50–83 (2011).

3. Dan S, Okamura M, Seki M, Yamazaki K, Sugita H, Okui M, Mukai Y, Nishimura H, Asaka R, Nomura K, Ishikawa Y, and Yamori T. Correlating phosphatidylinositol 3-kinase inhibitor efficacy with signaling pathway status: in silico and biological evaluations. *Cancer Research*, **70**(12):4982–4994 (2010).

4. Dinkel H, Michael S, Weatheritt RJ, Davey NE, Van Roey K, Altenberg B, Toedt G, Uyar B, Seiler M, Budd A, Jödicke L, Dammert MA, Schroeter C, Hammer M, Schmidt T, Jehl P, McGuigan C, Dymecka M, Chica C, Luck K, Via A, Chatr-Aryamontri A, Haslam N, Grebnev G, Edwards RJ, Steinmetz MO, Meiselbach H, Diella F, Gibson TJ. ELM-the database of eukaryotic linear motifs. *Nucleic Acids Research* **40**(Database issue):D242-51 (2012).

5. Engelman J A. Targeting PI3K signalling in cancer: opportunities, challenges and limitations. *Nature Reviews Cancer* **9**(8):550–562 (2009).

6. Fenton T R and Gout I T. Functions and regulation of the 70kDa ribosomal s6 kinases. *The International Journal of Biochemistry & Cell Biology* **43**(1): 47–59 (2011).

7. Franke T F. PI3K/Akt: getting it right matters. *Oncogene* **27**(50):6473–6488 (2008).

8. Fyffe C and Falasca M. 3-phosphoinositide-dependent protein kinase-1 as an emerging target in the management of breast cancer. *Cancer Management and Research* **5**:271–280 (2013).

9. Guo J-P, Coppola D, and Cheng J Q. IKBKE protein activates akt independent of phosphatidylinositol 3-kinase/PDK1/mTORC2 and the pleckstrin homology domain to sustain malignant transformation. *The Journal of Biological Chemistry* **286**(43):37389–37398 (2011).

10. Hancock C N, Macias A, Lee E K, Yu S Y, Mackerell A D Jr, and Shapiro P. Identification of novel extracellular signal-regulated kinase docking domain in- hibitors. *Journal of Medicinal Chemistry* **48**(14):4586–4595 (2005).

11. Hers I, Vincent E E, and Tavare J M. Akt signalling in health and disease. *Cellular Signalling* **23**(10):1515–1527 (2011).

12. PV Hornbec, JM Kornhauser JS Tkachev, B Zhang, E Skrzypek, B Murray, V Latham, M Sullivan. PhosphoSitePlus: a comprehensive resource for investigating the structure and function of experimentally determined post-translational modifications in man and mouse. *Nucleic Acids Research* **40**(Database issue):D261-70 (2012).

13. Hsu PP, Kang SA, Rameseder J, Zhang Y, Ottina KA, Lim D, Peterson TR, Choi Y, Gray NS, Yaffe MB, Marto JA, Sabatini DM. The mTOR-regulated phosphoproteome reveals a mechanism of mTORC1-mediated inhibition of growth factor signaling. *Science* **332**: 1317 (2011).

14. Janku F, Wheler J J, Westin S N, Moulder S L, Naing A, Tsimberidou A M, Fu S, Falchook G S, Hong D S, Garrido-Laguna I, Luthra R, Lee J J, Lu K H, and Kurzrock R. PI3K/AKT/mTOR inhibitors in patients with breast and gynecologic malignancies harboring PIK3CA mutations. *Journal of Clinical Oncology* **30**(8):777–782 (2012).

15. Laplante M and DM Sabatini DM. mTOR signaling in growth control and disease. *Cell* **149**(2):274–293 (2012).

16. Li X, Wilmanns M, Thornton J, Köhn M. Elucidating human phosphatase-substrate networks. *Science Signaling* **6**(275):rs10 (2013).

17. Linding R, Jensen LJ, Pasculescu A, Olhovsky M, Colwill K, Bork P, Yaffe MB, Pawson T. NetworKIN: a resource for exploring cellular phosphorylation networks. *Nucleic Acids Research* **36**(Database issue):D695-9 (2008).

18. Little A S, Smith P D, and Cook S J. Mechanisms of acquired resistance to ERK1/2 pathway inhibitors. *Oncogene* **32**(10):1207–1215 (2013).

19. Miller T W, Rexer B N, Garrett J T, and Arteaga C L. Mutations in the phosphatidylinositol 3-kinase pathway: role in tumor progression and therapeutic implications in breast cancer. *Breast Cancer Research* **13**(6):224 (2011).

20. Peri S, Navarro JD, Kristiansen TZ, Amanchy R, Surendranath V, Muthusamy B, Gandhi TK, Chandrika KN, Deshpande N, Suresh S, Rashmi BP, Shanker K, Padma N, Niranjan V, Harsha HC, Alreja TN, Vrushabendra BM, Ramya MA, Yatish AJ, Joy M, Shivashankar HN, Kavitha MP, Menezes M, Choudhury DR, Ghosh N, Saravana R, Chandran S, Mohan S, Onnalagadda JCK, Prasad CK, Kumar-Sinha C, Deshpande KS, Pandey A. Human protein reference database as a discovery resource for proteomics. *Nucleic Acids Research* **32**(Database issue):D497-501 (2004).

21. Roberts P J and Der C J. Targeting the raf-MEK-ERK mitogen-activated protein kinase cascade for the treatment of cancer. *Oncogene* **26**(22):3291–3310 (2007).

22. Wilkes EH, Terfve C, Gribben JG, Saez-Rodriguez J and Cutillas PR. Empirical inference of circuitry and plasticity in a kinase signalling network. *Proc. Nat. Acad. Sci. USA* **112**(25):7719-24 (2015).
